# Contrasting host–pathogen interactions and genome evolution in two generalist and specialist microsporidian pathogens of mosquitoes

**DOI:** 10.1038/ncomms8121

**Published:** 2015-05-13

**Authors:** Christopher A. Desjardins, Neil D. Sanscrainte, Jonathan M. Goldberg, David Heiman, Sarah Young, Qiandong Zeng, Hiten D. Madhani, James J. Becnel, Christina A Cuomo

**Affiliations:** 1 Broad Institute of MIT and Harvard, Cambridge, Massachusetts 02142, USA; 2 USDA, ARS, Center for Medical, Agricultural and Veterinary Entomology, 1600 SW 23rd Drive, Gainesville, Florida 32608, USA; 3 Department of Biochemistry and Biophysics, University of California-San Francisco, San Francisco, California 94158, USA

## Abstract

Obligate intracellular pathogens depend on their host for growth yet must also evade detection by host defenses. Here we investigate host adaptation in two Microsporidia, the specialist *Edhazardia aedis* and the generalist *Vavraia culicis*, pathogens of disease vector mosquitoes. Genomic analysis and deep RNA-Seq across infection time courses reveal fundamental differences between these pathogens. *E. aedis* retains enhanced cell surface modification and signalling capacity, upregulating protein trafficking and secretion dynamically during infection. *V. culicis* is less dependent on its host for basic metabolites and retains a subset of spliceosomal components, with a transcriptome broadly focused on growth and replication. Transcriptional profiling of mosquito immune responses reveals that response to infection by *E. aedis* differs dramatically depending on the mode of infection, and that antimicrobial defensins may play a general role in mosquito defense against Microsporidia. This analysis illuminates fundamentally different evolutionary paths and host interplay of specialist and generalist pathogens.

## Abstract (web summary)

Microsporidia are intracellular parasitic fungi that infect diverse animal hosts including humans. Here, Desjardins *et al.* present genomic and transcriptomic data for two microsporidia that infect disease-transmitting mosquitoes, highlighting differences in potential host interplay mechanisms.

## Graphical abstract

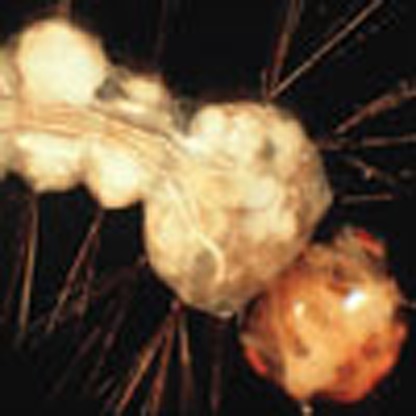

Obligate intracellular pathogens rely on a complex interplay with their hosts; they must acquire nutrients and other metabolites within the host cell, yet evade its defenses. Microsporidia, recently called ‘The Master Parasites’^1^, are obligate intracellular fungal pathogens that evolved from free-living fungal ancestors and infect a broad range of hosts including humans and most other animals. The infective stage of Microsporidia in the spore includes a highly developed injection apparatus used to penetrate the host cell (Fig. 1a,b). These pathogens then undergo most of their development within the host cytoplasm, which allows direct transport of nutrients and energy from the host to the pathogen. However, intracellular growth may also expose the pathogen to attack by the host cellular immune system.

Microsporidia are noted for having compact genomes with reduced metabolic capacity, highlighting host dependencies of these intracellular pathogens. Genome size is typically small, in the range of 2.3–9 Mb; however, substantially larger genomes have also been described^2^. In addition, genes are unusually compacted, with very few introns and small intergenic regions, as shown for *Encephalitizoon cuniculi*^3^. Multiple studies have revealed loss of essential metabolic pathways, including *de novo* synthesis pathways for amino acids, nucleotides and even ATP, all of which must in turn be acquired from the host^3^,^4^,^5^. Microsporidia have evolved many mechanisms to acquire these nutrients, including ATP transporters used to ‘steal’ ATP from host cells^6^,^7^. Recent studies have shown that some Microsporidia secrete hexokinase^8^,^9^ and trehalase^9^, which may drive production of basic metabolites by the host for acquisition by the Microsporidia.

Microsporidian genes involved in other aspects of host–pathogen interactions, such as evasion of the host immune response, are not well characterized. Secreted proteins may include effectors that interact with host cell proteins to favour pathogen growth^10^. Despite widespread gene loss, microsporidian genomes encode many expanded gene families, particularly species-specific proteins with secretion signals, hypothesized to be involved in these host–pathogen interactions^5^,^8^,^11^,^12^. In addition, unknown is how nutrient acquisition, immune evasion and other interactions may differ in specialist versus generalist pathogens.

Here we investigate genome reduction and host–pathogen interactions in two Microsporidia, one specializing on a single host and one infecting a wider range of species, that naturally infect major disease vector mosquitoes. *Edhazardia aedis* is a pathogen of the mosquito *Aedes aegypti*, a major disease vector that transmits dengue haemorrhagic fever, yellow fever and chikungunya. *E. aedis* is specific to *Ae. aegypti* and has exploited virtually every life stage and tissue of its host in a complicated life cycle involving two generations of the host (Fig. 1c,d)^13^,^14^. *Vavraia culicis* is another microsporidian pathogen of mosquitoes, although unlike *E. aedis* it infects a wide range of mosquito species. Species of *Culex*, the major disease vector of West Nile virus, and species of *Anopheles*, the major disease vector of malaria, are particularly susceptible. Relative to *E. aedis*, *V. culicis* has a simple life cycle (Fig. 1e).

We describe the genome sequence of *E. aedis* and *V. culicis*, and identify the key differences in these species that resulted from genome contraction under differing selective pressures. To illuminate changes in both pathogen and host transcriptomes over the course of infection, we deeply sequence RNA from infected and uninfected host tissues throughout both pathogens’ life cycles. This combination of genomic and transcriptomic analyses reveals how both host and pathogen have adapted to these different models of infection.

## Results

### Genome variation and impact of low GC content

**Figure 1:**
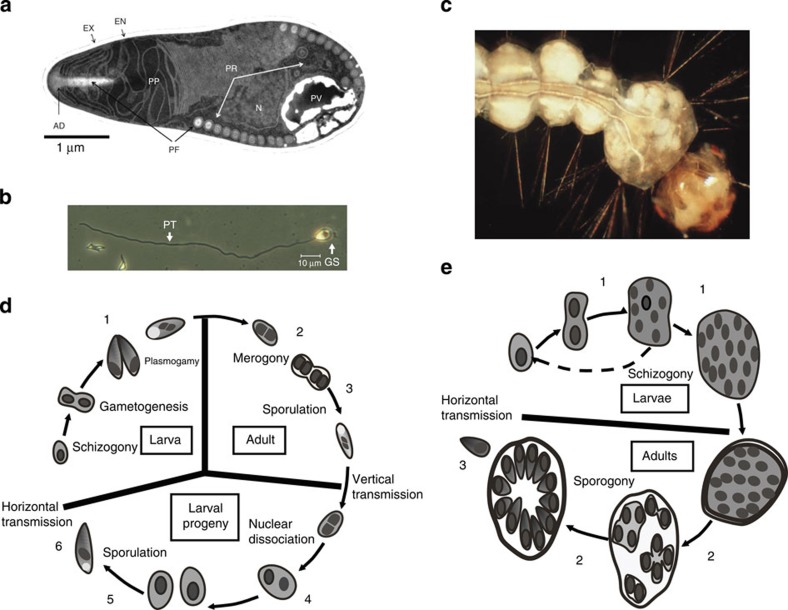
Life cycles of *E. aedis* and *V. culicis*. (**a**) Transmission electron micrograph of a mature microsporidian spore demonstrating the complex injection apparatus. The spore wall is composed of an exospore (EX) and endospore (EN) that contains chitin. The injection apparatus is a three part complex with a polar filament (PF) that is attached at the anterior end by the anchoring disk (AD), the membranous polaroplast (PP) and the posterior vacuole (PV). The nucleus (N) is centrally located and much of the volume of the spore is occupied by polyribosomes (PR). (**b**) A light micrograph of a germinated spore (GS) with the everted polar filament to become the polar tube (PT), through which the germ cell (sporoplasm) is injected into the host cell. (**c**) A fourth instar larva of *Ae. aegypti* infected by *E. aedis* via vertical transmission, corresponding to sample 5 in part D. Masses of spores in fat body (white cysts) are released on death to initiate horizontal infection in a new host generation. (**d**) Life cycle of *E. aedis*, modified from ref. 80. The six time points for RNA-Seq samples are indicated with numbers. *E. aedis* spores in the environment are horizontally transmitted when ingested by larval mosquitoes and then aggregate in the gastric caeca (a specialized region of the midgut), where they develop into the first intracellular spores (1). These spores penetrate the midgut wall and invade host cells called oenocytes (2) and following a blood meal by the adult mosquito they develop into the second intracellular spores and migrate to the ovaries (3). These spores are then vertically transmitted to the next generation of larvae as they develop in fat body (4) and undergo meiosis and sporulation (5) to produce spores to be released into the environment (6). (**e**) Life cycle of *V. culicis*, modified from ref. 80. The three time points for RNA-Seq samples are indicated with numbers. Spores in the environment are ingested by larvae, penetrate the midgut wall and systemically infect the host as it develops from larva (1) to adult (2), where *V. culicis* produces new spores to be released into the environment (3).

We sequenced, assembled and annotated the genomes of both *E. aedis* and *V. culicis*, and found that they exhibit a nearly 10-fold variation in genome size but only a 1.5-fold variation in gene content (Table 1). Genes predicted for each assembly were supported by RNA-Seq of samples representing each of the major life cycle stages of each species (Methods, Fig. 1d,e). While the genome of *E. aedis* is more fragmented than that of *V. culicis*, both genomes have similar coverage of a core eukaryotic gene set^15^ as other Microsporidia (Supplementary Fig. 1), suggesting that both assemblies contain nearly complete gene sets. The 51.3-Mb assembly of the *E. aedis* genome represents the largest sequenced microsporidian genome to date, substantially larger than the 23–24 Mb estimated size of *Octosporea bayeri*^2^ and *Anncaliia algerae*^16^. By comparison, the 6.1-Mb *V. culicis* genome assembly has a more typical size, GC content and coding capacity for the Microsporidia. Both the *E. aedis* and *V. culicis* genomes encode RNA interference (RNAi) machinery and, notably, genomes encoding RNAi were significantly larger than those that did not (*P*<0.006; Mann–Whitney *U*-test, Supplementary Fig. 2).

The expanded genome size of *E. aedis* is not due to a higher fraction of repetitive sequence. Only 5.5% of the assembly is repetitive sequence, and of this only 222 kb could be classified, matching either LINE or gypsy transposable elements (Methods). Instead, the larger genome size resulted primarily from expanded AT-rich intergenic regions (Supplementary Note 1, Supplementary Fig. 3); overall, the genome is 78% AT and only 9% of sequence is coding, similar to the estimates from a small-scale survey of 233.5 kb of *E. aedis*^17^. The extreme AT-bias of the *E. aedis* genome extends to coding regions, where it has influenced both codon and amino-acid usage (Supplementary Note 1, Supplementary Figs 4 and 5).

The genomes of both *V. culicis* and *E. aedis* are heterozygous, although the level of variation is substantially different between these species. The *V. culicis* genome contains one single-nucleotide polymorphism (SNP) every 700 bases, with an average allele balance of 58% (Methods). A higher number and frequency of SNPs were found in *E. aedis*, with one SNP every 284 bases and an average allele balance of 59%. The observation of heterozygous positions across the genome, as also observed in some other Microsporidia species^8^,^18^,^19^, suggests that both *V. culicis* and *E. aedis* are also diploid.

### Refined phylogeny and differential metabolic pathway loss

**Figure 2:**
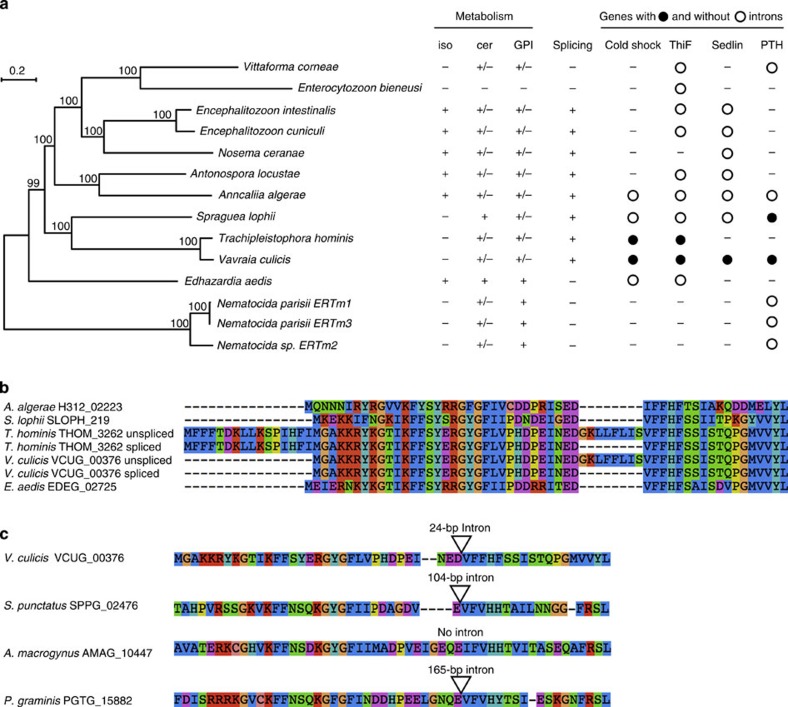
Phylogeny of Microsporidia and conservation of introns. (**a**) Phylogeny of Microsporidia based on 217 single-copy core genes. The phylogeny was estimated using RAxML^70^ and a PROTCATLG model of evolution. Bootstrap support (BS) is shown above each node. The presence of complete (+) or partial (+/−) pathways is shown for isoprenoid synthesis (iso), ceramide synthesis (cer) and GPI. Also shown is the phylogenetic profile for the splicesomal machinery, along with individual profiles for the four spliced genes with known PFAM domains predicted in the *V. culicis* genome (open circle: present with intron; closed circle: present without intron). (**b**) Peptide alignment of the first 75 amino acids of the cold shock domain-containing gene, showing for *V. culicis* and *T. hominis* both the unspliced and spliced forms. The intron in these species clearly represents a region absent from the gene in other Microsporidia. (**c**) Peptide alignment of the region of the cold shock domain-containing gene containing the intron between *V. culicis*, chytrid fungi *Spizellomyces punctatus* and *Allomyces macrogynus*, and the basidiomycete fungus *Puccinia graminis*. Both *S. punctatus* and *P. graminis* have an intron inserted at a position identical to *V. culicis*.

To estimate the phylogenetic position of these species, orthologues were identified between *E. aedis, V. culicis* and 12 additional Microsporidia (Methods, Supplementary Fig. 2). The estimated phylogeny (Fig. 2a, Supplementary Fig. 2) is concordant with previous phylogenies examining a subset of species^8^,^20^. *V. culicis* was found to be more closely related to the human pathogen *Trachipleistophora hominis* (0.169 changes per site) than species within the genera *Nematocida* (0.268 changes per site) and *Encephalitozoon* (0.205 changes per site). *E. aedis* was predicted to be the next most basal Microsporidia to *Nematocida* (Supplementary Note 2). While *E. aedis* has the largest predicted number of protein-coding genes in the Microsporidia compared here, this does not reflect higher retention of genes conserved with other species, but rather genes unique to *E. aedis* (Supplementary Fig. 2), including 897 paralogous genes in 180 expanded gene families. As previously shown in *Encephalitizoon cuniculi*, *Enterocytozoon bienusi* and *Octospora bayeri*^2^,^3^, analysis of core eukaryotic genes^15^ revealed that in Microsporidia these conserved genes have shorter coding sequences than their fungal orthologues (Supplementary Fig. 6, Supplementary Note 3).

Utilizing the phylogenetic tree and orthologues, we identified genes differentially lost or retained in *E. aedis* and *V. culicis* (Fig. 2a, Supplementary Note 4). The *V. culicis* genome encodes a number of basic metabolic genes not encoded by the *E. aedis* genome involved in folate metabolism (*DFR1*, *SHM* and *CDC21*) and in synthesis and interconversion of pyrimidines (*URA6*, *URA7* and *CDC21*). This suggests that *E. aedis* is more heavily reliant on its host for these resources. The *E. aedis* genome encodes pathways that produce cell membrane components that are either lost or reduced in the *V. culicis* genome. These include an isoprenoid biosynthesis pathway that terminates at farnesyl diphosphate, a precursor to the production of sterols (including ergosterol, a major component of fungal cell membranes and a target of antifungal drugs), and all genes necessary for glycosylphosphatidylinositol (GPI) anchor biosynthesis, allowing it to tether proteins to the cell membrane. *E. aedis* has also retained the ability to produce ceramides through a partial sphingolipid biosynthesis pathway. Although the exact function of this pathway in Microsporidia is unknown, many intermediates of the sphingolipid biosynthesis pathway act as signalling molecules^21^ or immunoregulators (for example, in *Talaromyces*^22^). The pathway for isoprenoid synthesis is completely lost in *V. culicis*, while the GPI anchor and ceramide pathways are heavily reduced (Supplementary Note 4).

Orthologue profiles were also used to identify genes potentially involved in host–pathogen interactions. Both the *E. aedis* and *V. culicis* genomes contain one copy of hexokinase (EDEG_00794, VCUG_00438) and one or two copies of trehalase, respectively (EDEG_03004, VCUG_00959 and VCUG_00974) with a predicted secretion signal; when secreted these factors could drive host metabolism to the advantage of the Microsporidia, as suggested for hexokinase^8^,^9^. Furthermore, the *E. aedis* and *V. culicis* genomes encode secreted ubiquitin hydrolases, which could provide resistance to the ubiquitin system, recently implicated in immune defense against Microsporidia in *Caenorhabditis elegans*^23^. We also identified 335 and 169 secreted proteins in the *E. aedis* and *V. culicis* genomes, respectively, that are expressed during infection but have no predicted domains; most of the proteins also appear species-specific (Table 1, Methods). These secreted proteins could include candidate effectors that interact with the host.

### Phylogenetic profiling of splicing proteins

Variable loss of introns and genes encoding components of the spliceosome has been previously noted in other microsporidian genome analyses^4^,^11^. On the basis of motif searches and genomic alignments of RNA-Seq reads (Methods), we identified introns in 27 genes in *V. culicis* (Supplementary Table 1, Supplementary Note 5). Most were inefficiently spliced (median efficiency, 25.3%), as in *En. cuniculi*^24^, suggesting that this is a general feature of spliced genes in Microsporidia. No introns were identified in *E. aedis*; splicing motifs were absent from the genome and no spliced alignments were detected with RNA-Seq data. The *V. culicis* genome also includes components of the spliceosome missing in *E. aedis*, described below. Phylogenomic comparisons (Fig. 2a) suggest that canonical splicing was lost or reduced at least three times over the course of microsporidian evolution—in *E. aedis*, *Nematocida* and the ancestor of *Ent. bieneusi* and *V. corneae*.

Given these independent losses, we utilized the broader taxon sampling of Microsporidia genomes to identify core components of the microsporidian splicing machinery. Using orthologues of 89 components involved in splicing in *Saccharomyces cerevisiae*, we examined the conservation pattern across Microsporidia (Supplementary Data 1). In the five genomes for which canonical splicing has not been detected, fewer components were conserved; Microsporidia with splicing contain between 17 and 31 (average of 22.5) splicing components, whereas species without splicing contain between 5 and 10 (average of 8.5). Orthologues of four genes were present in most species without splicing, including a Sm protein (SmB), two U5 small nuclear ribonucleoprotein particle (snRNP) components (Prp8 and Brr2) and a RNA-binding component of the U4/U6 snRNP (Snu13). Microsporidia-specific splicing factors may substitute for some components; phylogenetic profiling identified 10 proteins conserved specifically in Microsporidia with splicing, but without a known function (Supplementary Data 2, Supplementary Note 6).

We identified four spliced genes in the *V. culicis* genome with known functions that were not ribosomal proteins. Each was present in Microsporidia species with and without splicing (Fig. 2a); however, introns in these genes were only found in *V. culicis* and close relatives, even for genes present in other Microsporidia capable of splicing. The four genes are functionally diverse based on the protein families (PFAM) domains found in each for cold shock, ThiF, Sedlin N and peptidyl-tRNA hydrolase. Alignment of the cold shock protein sequences highlights that the exact region spliced out by the intron in *V. culicis* and *T. hominis* is missing from that protein in other species (Fig. 2b). Orthologue clustering of Microsporidia with 12 other fungi (Methods) showed that this gene is present in three divergent fungal species, of which two have an intron inserted at a position identical to *V. culicis* (Fig. 2c), suggesting that this intron was present in the ancestor of these species. The simplest explanation for this pattern is widespread intron loss across Microsporidia, leaving a small number of introns in species that have retained substantial splicing machinery.

### Dynamic changes in *E. aedis* transcriptome during infection

**Figure 3:**
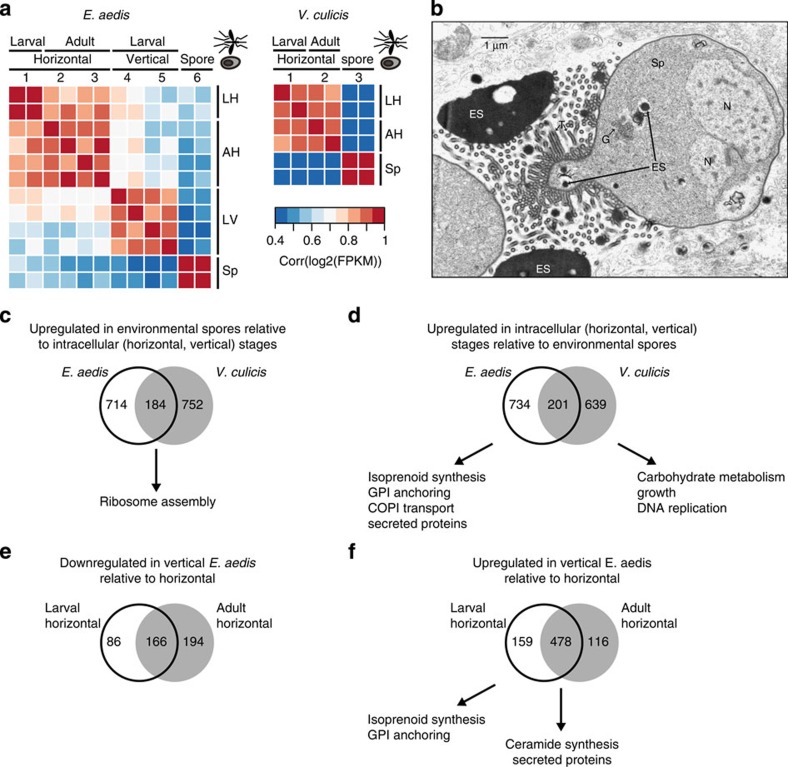
Transcriptional signatures of *E. aedis* and *V. culicis* life cycles. (**a**) Pairwise correlation coefficients of transcript abundances between samples of *E. aedis* and *V. culicis*. Coefficients were calculated from log2-transformed FPKM values. Numbers correspond to time points in Fig. 1d,e, and the position of the sample in both the microsporidian and mosquito lifecycle is shown on the *x* axis. Letter codes on the *y* axis correspond to labels on the *x* axis: LH: larval horizontal; AH: adult horizontal; LV: larval vertical; Sp: environmental spores. (**b**) Transmission electron micrograph of a diplokaryotic sporont (Sp) of *E. aedis* in the vertically transmitted portion of the life cycle. Electron-dense secretions (ES) in the cytoplasm are transported into the episporontal lumen via tubules (T) where they accumulate. G (Golgi bodies); N (Nucleus). (**c**–**f**) Venn diagrams of differentially expressed genes (*q*<0.05), with enriched functional classes (**c**) upregulated in environmental spores of *E. aedis* and *V. culicis* relative to intracellular stages; (**d**) upregulated in intracellular stages of *E. aedis* and *V. culicis* relative to environmental spores; (**e**) downregulated in the vertically transmitted portion of the *E. aedis* life cycle relative to the horizontally transmitted portion; (**f**) upregulated in the vertically transmitted portion of the *E. aedis* life cycle relative to the horizontally transmitted portion.

To identify transcriptional signatures of the life cycles of these Microsporidia, gene expression was quantified from time points representing the major stages of infection, with two independent samples per time point shown in Fig. 1d,e (Supplementary Data 3 and 4). Correlations of expression values between samples revealed that *E. aedis* transcription was grouped into three main clusters: environmental spores, horizontally transmitted intracellular stages and vertically transmitted intracellular stages (Fig. 3a). Similarly, *V. culicis* transcription formed two main clusters: environmental spores and horizontally transmitted intracellular stages (Fig. 3a), with no apparent additional clustering by host life stage.

To investigate the genes driving these patterns, we identified differentially expressed gene clusters in *E. aedis* and *V. culicis*, and then evaluated enrichment of Gene Ontology (GO) terms, metabolic pathways and secreted proteins of unknown function (Fig. 3c–f). Within the environmental spore clusters in both *V. culicis* and *E. aedis*, most enriched GO terms were involved in ribosome assembly (Supplementary Tables 2 and 3, Fig. 3c), consistent with the large number of polyribosomes observed in environmental spores (Fig. 1a). In addition to ribosomal proteins, Hsp70 domain proteins potentially involved in protein folding are among the mostly highly expressed genes in *E. aedis* (EDEG_00132, EDEG_00133), as previously noted in a survey of spore-expressed sequence tags^25^, as well as in *V. culicis* (VCUG_01919). This may be a reflection of environmental spores representing a relatively passive life stage focused on basic conserved cellular processes rather than expending resources to actively affect the host.

Evaluation of genes upregulated in intracellular stages of *E. aedis* and *V. culicis* showed that in contrast to spores, there was little overlap in GO term enrichment between the two species in developmental stages (Fig. 3d, Supplementary Tables 4 and 5). Genes upregulated in *V. culicis* were enriched for GO terms representing growth, carbohydrate metabolism and DNA replication. By contrast, genes upregulated in *E. aedis* were enriched for pathways involved in protein modification and trafficking, such as isoprenoid synthesis, GPI anchoring and COPI (coat protein) synthesis, which is involved in Golgi–ER transport. Gene-level examination of these pathways supported these findings (Supplementary Note 7). The intracellular stages of *E. aedis* were also significantly enriched for secreted proteins of unknown function, although the intracellular stages of *V. culicis* were not (*P*<1e−5 and *P*<0.24, respectively; *χ*^2^-test). These patterns suggest that the generalist *V. culicis* intracellular stages are simpler than the specialist *E. aedis* intracellular stages, focused on growth rather than protein modification and trafficking, and with fewer surface decorations and secreted proteins that could interact with the host.

Within the intracellular stages of *E. aedis*, genes specifically upregulated within the horizontal (time points 1–3) or vertical (time points 4 and 5) portion of the life cycle revealed a shift in metabolic potential as well as in secreted proteins that could mediate host interactions (Fig. 3e,f). GO enrichment analysis of genes upregulated during the vertical portion revealed terms related to lipid synthesis (Fig. 3f, Supplementary Tables 6 and 7), including GPI anchor, isoprenoid and ceramide synthesis (Supplementary Fig. 7). A significantly greater number of secreted proteins of unknown function were also upregulated during this period than the horizontal period (*P*<4e−6; *χ*^2^-test; Fig. 3f), including three expanded gene families (Supplementary Fig. 8). These findings are corroborated by the observation that secreted proteins are produced in such abundance during the vertical portion of the life cycle that they are easily visible in micrographs (Fig. 3b), suggesting that protein modification and trafficking are particularly important during this portion of the *E. aedis* life cycle. By contrast, the horizontal portion of the life cycle showed no enrichment of GO terms (Fig. 3e). However, a comparison of time points pre- (2) and post-bloodmeal (3) revealed that the secreted hexokinase and trehalase were both significantly upregulated following a bloodmeal (*q*<0.0027 and *q*<0.0038, respectively, Supplementary Data 3).

### Expression of meiotic genes during *E. aedis* sexual cycle

Given that *E. aedis* has a well-defined sexual cycle, whereas meiosis has not been observed in *V. culicis*, we evaluated stage-specific expression of fungal meiotic genes in our transcriptome time course. We first identified a candidate mating-type locus in each species that included a high-mobility-group transcription factor gene (EDEG_02959, VCUG_02536) that is highly expressed across all time points. The synteny of genes flanking the HMG in some Microsporidia species^26^ (but notably not in *T. hominis*^27^), a RNA helicase and a triose phosphate transporter (TPT), is partially observed in these species; each locus has one of the two flanking genes, the RNA helicase in *E. aedis* or the TPT in *V. culicis*, while the other gene is found on a different scaffold in each assembly. Of a set of 12 conserved genes important for meiotic recombination^28^, 9 are conserved in *E. aedis* and 10 in *V. culicis*; other Microsporidia contain homologues of 5–10 of these genes (Supplementary Data 5).

We next examined expression of these meiotic genes (Supplementary Data 3 and 5) to correlate their expression with the vertically transmitted intracellular stages in *E. aedis* when meiosis occurs. Genes significantly upregulated (*q*<0.05; negative binomial exact test^29^) during these stages included *SPO11*, *HOP1*, dimer-forming *MSH4* and *MSH5*, and one of two copies of *MND1* (EDEG_03123). By contrast, *HOP2*, the other copy of *MND1* (EDEG_01364), and *RAD51* were expressed in all intracellular stages and environmental spores. *RAD51* is essential for repair of double-strand breaks in other stages in addition to meiosis. As *HOP2* and *MND1* are involved in meiotic chromosome synapsis in *Saccharomyces cerevisiae,* their expression should be specific to the vertical stage of *E. aedis,* the only stage where meiosis is known to occur. The significant upregulation (*q*<0.05) of these genes in spores of both species suggests that they may have an alternate function in this stage of Microsporidia. All nine identified meiotic genes in *E. aedis* were expressed during at least one life stage, while in *V. culicis SPO11* and *MSH4* were present but not expressed in any stage.

### Host immune response to *E. aedis* infection

To evaluate the effect of *E. aedis* infection on *Ae. aegypti* transcription, *Ae. aegypti* gene expression was calculated for infected and control samples at each time point shown in Fig. 1d (Supplementary Data 6). Differentially expressed mosquito genes between control and infected samples at each time point were identified (Supplementary Table 8) and GO term and KEGG pathway enrichment were evaluated (Table 2, Supplementary Table 9). Early during infection, when *E. aedis* invades the midgut (time point 1), alkaline phosphatase activity was downregulated in infected samples (Table 2). The same effect was observed in the midguts of *Nosema*-infected bees^30^, where the authors hypothesized this resulted in a reduction in gut protection and health. Iron homeostasis and transport was upregulated in infected samples during midgut invasion; this result appeared to be driven by an increased expression of the iron storage and transport genes ferritin and transferrin. Iron is critical to both the host immune response and pathogen growth, although the interplay between these two factors can be complex^31^,^32^.

Both during midgut infection and the subsequent stages when *E. aedis* occupies *Ae. aegypti* oenocytes and migrates to the ovaries (time points 2 and 3), immune-response-related GO terms were enriched in infected samples, including serine endopeptidase activity, lysozyme activity and rRNA N-glycosylase activity (Table 2). The genes mapping to rRNA N-glycosylase activity share sequence similarity to ribosome-inactivating proteins, which are used by plants in various immune responses^33^. Evidence of a stress response is also detected in time point 2; two *HSP70* genes are induced more than sevenfold in infected mosquitoes, and are among the top seven most highly induced genes in this stage.

**Figure 4:**
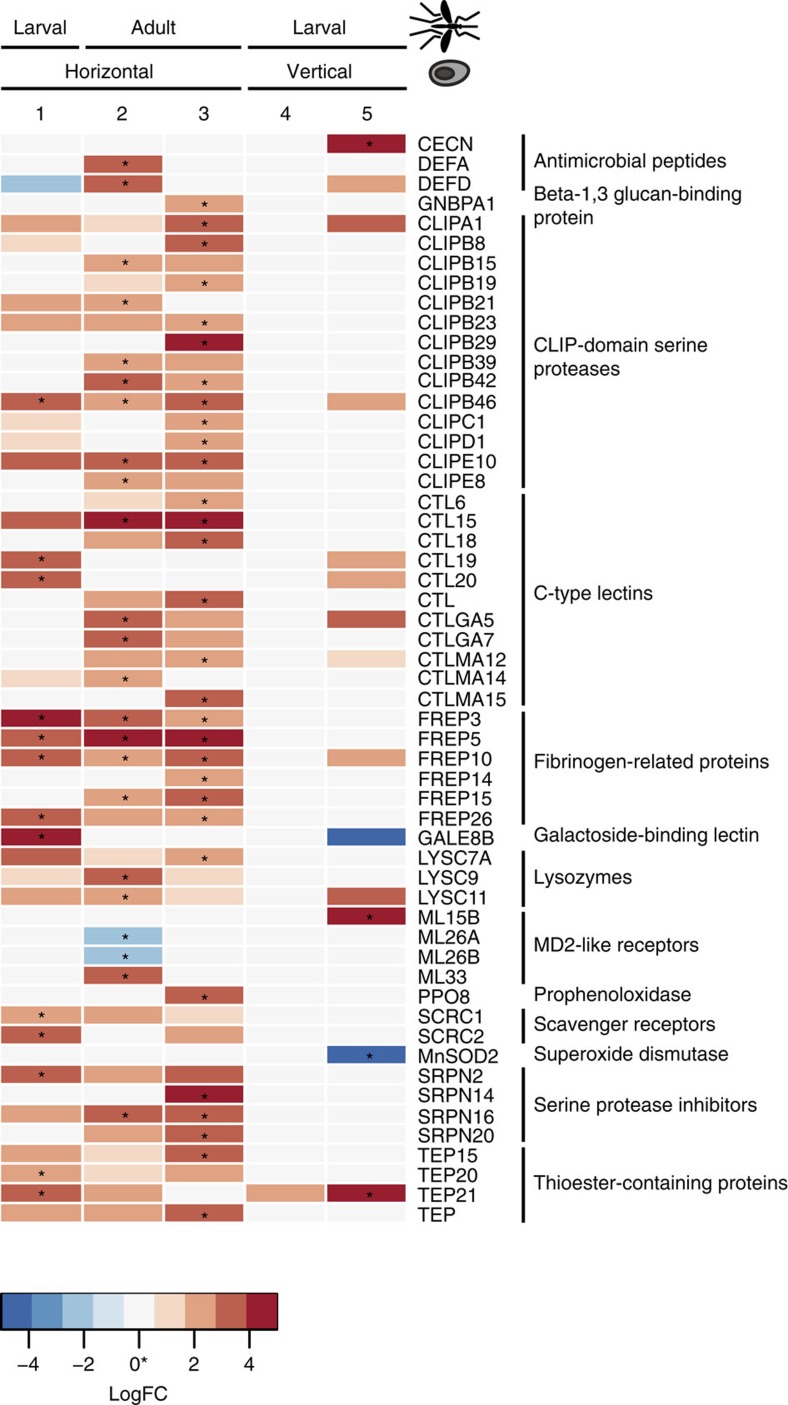
Differentially expressed immunity genes in infected *Ae. aegypti*. Boxes marked with asterisks represent genes that were significantly differentially expressed at *q*<0.05; coloured boxes represent genes that were differentially expressed at *P*<0.05. Red boxes represent genes upregulated during infection, while blue boxes represent genes downregulated during infection. Numbers correspond to time points in Fig. 1b, and the position of the sample in both the microsporidian and mosquito lifecycle is shown on the *x* axis.

To more specifically evaluate this immune response, we classified differentially expressed genes involved in mosquito immunity using ImmunoDB^34^ (Fig. 4). Numerous fibrinogen-related proteins that are hypothesized to act in pathogen recognition in mosquitoes^35^ were strongly upregulated in infected mosquitoes in all of the first three time points. While not included in ImmunoDB, an alpha-integrin, which can serve as a receptor for fibrinogen, was significantly upregulated during the first phase of infection (AAEL014660, *q*<0.0001). Other classes of upregulated genes include the antimicrobial peptides (defensin A and D), CLIP-domain serine proteases, C-type lectins, lysozymes, serine protease inhibitors and thioester-containing proteins, some of which were identified through the GO term enrichment analysis. However, after vertical transmission of *E. aedis* to the second host generation (time points 4 and 5), little GO term enrichment or differential expression of immune-related genes was observed (Table 2, Fig. 4), suggesting that little immune response is mounted by the mosquito against the inherited portion of the infection.

Given that expression of the *E. aedis* secreted hexokinase (EDEG_00794) and trehalase (EDEG_03004) increased following the bloodmeal (from time point 2–3; Supplementary Data 3), we examined expression of glycolytic enzymes in *Ae. aegypti* at these time points (Supplementary Data 6 and Supplementary Table 10). While expression of most glycolytic enzymes was reduced following the bloodmeal in both control and infected mosquitoes, expression levels dropped significantly less in infected mosquitoes (*P*<0.03; Wilcoxon-signed-rank test). Together, these upregulated *E. aedis* and mosquito genes suggest a connection between pathogen and host metabolism.

### *V. culicis* infection affects muscle and defensin expression

**Figure 5:**
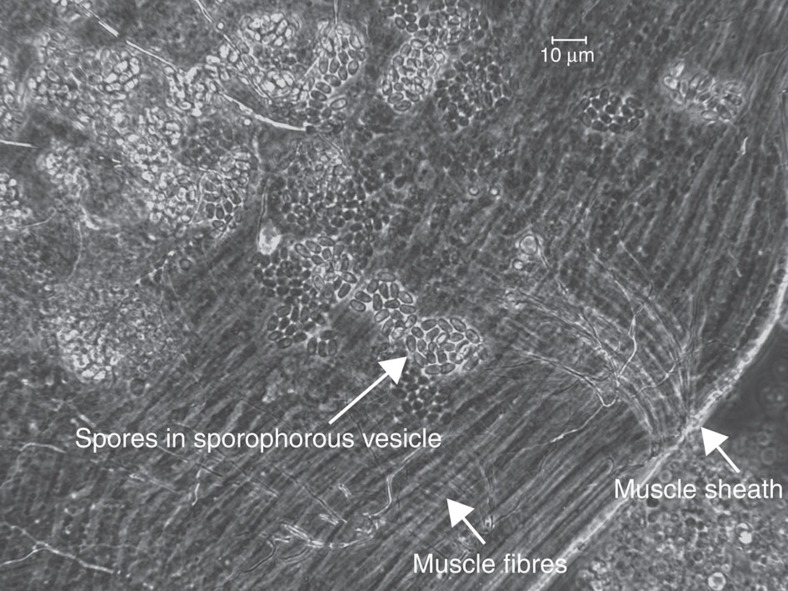
Spores of *Vavraia culicis* infecting muscle in the thorax of an adult *Anopheles quadrimaculatus*. Groups of spores are contained within a sporophorous vesicle and localized to fibers limited by the muscle sheath.

As described for *Ae. aegypti*, gene expression was calculated for infected and control samples for *An. quadrimaculatus* at each time point as shown in Fig. 1e (Supplementary Data 7), differentially expressed genes were identified (Supplementary Table 8) and GO term and KEGG pathway enrichment were evaluated (Table 2, Supplementary Table 9). *V. culicis* undergoes a simpler life cycle than *E. aedis* in which environmental spores infect a single generation of host. In infected host larvae, there were no enriched GO terms and very few differentially expressed genes overall (Supplementary Table 8). In adult infected samples, the top five enriched GO terms all related to muscle contraction and assembly, as did all enriched KEGG pathways (Table 2, Supplementary Table 9). *V. culicis* causes systemic infections in adult mosquitoes, with flight muscles a primary site of infection^36^ (Fig. 5). Furthermore, many species of closely related genera (*Vavraia*, *Trachipleistophora* and *Pleistophora*) were originally isolated from muscle tissue^37^,^38^,^39^,^40^,^41^. If infection is concentrated in the muscle tissue, transcription in that tissue could be modified in response to or driven by the infection.

In contrast to *Ae. aegypti*, when *An. quadrimaculatus* genes were mapped using ImmunoDB^34^, the only differentially expressed immunity gene at any time point during infection (negative binomial exact test^29^, *q*<0.05) was an antimicrobial defensin (Supplementary Data 7). In the larval stage, defensin *DEF1* was significantly upregulated during infection (*q*<0.05), while in the adult stage it was also upregulated during infection, although not significantly (*P*<0.002, *q*<0.2). This suggests that the generalist *V. culicis* evades or suppresses the majority of the immune response of its mosquito host.

## Discussion

Here we present genomic analysis of two species of Microsporidia with similar hosts but very different host ranges and genome architecture. The large genome size of *E. aedis* contrasts not just with *V. culicis* but with other Microsporidia sequenced to date, and with the paradigm of small genome size being strongly selected for this group of obligate pathogens. The larger genome is due to an expansion of AT-rich intergenic regions and does not include a greater number of ancestrally conserved genes than other Microsporidia. Sequencing additional isolates of *E. aedis* would enable analysis of the current constraints on genome size, such as the overall and regional bias for insertions or deletions.

Phylogenomic profiling of components of the spliceosome across Microsporidia and identification of spliced transcripts through deep RNA-Seq highlight differences in splicing retention and loss in the Microsporidia. Many spliceosomal components conserved in fungi were not detected in any microsporidian genome, including those with spliced transcripts. Either these components are more highly divergent in Microsporidia or a more minimal spliceosome may have been selected for, perhaps adapted to the smaller number and size of introns in these species or by the relaxation of splicing efficiency. Furthermore, some splicing proteins were identified in all Microsporidia, including those without spliced transcripts. The retention of these proteins is intriguing and suggests they serve a functional role in Microsporidia, either to splice undetected or noncanonical introns or for some other alternative purpose.

Our analysis for the first time characterizes the meiotic gene expression programme in a Microsporidia species with a known sexual cycle. Both *E. aedis* and *V. culicis* have conserved a core set of meiotic genes and a candidate mating-type locus; as meiosis has only been observed in *E. aedis*, the presence of these genes in *V. culicis* suggests that it may be capable of meiosis under some alternate conditions, or that these genes have lost functionality in *V. culicis* but have not yet been purged from the genome. A subset of meiotic genes are indeed specifically induced during sexual stages of *E. aedis* and not detected in the sampled stages of *V. culicis*. The more ubiquitous expression of *MND1*, involved in meiotic recombination, suggests that this may play additional roles in Microsporidia, as suggested for mammals based on expression in nongermline tissues^42^.

This genomic analysis also sheds light on the basis of the complex interaction between the specialist *E. aedis* and its host. The *V. culicis* genome encodes a greater capacity for folate metabolism and pyrimidine interconversion than *E. aedis*, perhaps reflecting a generalist pathogen needing to be less reliant on its host than a specialist. However, numerous metabolic pathways involved in protein modification and trafficking are retained exclusively in the *E. aedis* genome, and these pathways are upregulated during later stages of infection. In addition, during these later stages, secreted protein complexes are so abundant as to be visible in micrographs (Fig. 3b). While the chemical nature of these secretions is poorly understood^43^, a few hypotheses have suggested a role in microsporidian metabolism or generation of a conductive pathway, based on the similarity of some secretions to cytoskeletal components^44^. In contrast, *V. culicis* transcription appears focused on growth and basic metabolic functions. This may reflect the adaptation of *E. aedis* to a specific host, whereas *V. culicis* has maintained a simpler lifecycle, allowing it to grow in a wider range of hosts.

Secreted proteins may not only deal with immune evasion, but also with manipulation of host metabolism. Following a blood meal in *Ae. aegypti*, the *E. aedis* secreted hexokinase and trehalase were both significantly upregulated, suggesting an increased control of host metabolism later in the horizontal portion of the life cycle, which is critical to ensure vertical transmission. Supporting this, while expression of most *Ae. aegypti* glycolytic enzymes was reduced following the bloodmeal in both control and infected mosquitoes, expression levels dropped significantly less in infected mosquitoes. It is possible that increased secretion of these enzymes by *E. aedis* mitigates a typical reduction in glycolytic activity in *Ae. aegypti* following a bloodmeal.

Both mosquitoes responded to microsporidian infection by inducing antimicrobial peptides. Defensins were upregulated in response to infection by both mosquitoes, suggesting that these antimicrobial peptides may be general elements of the mosquito defensive response against microsporidian pathogens. Cercopin A was induced by *Ae. aegypti* during later stages of infection; *E. aedis* invades the fat body, the site of cercopin production, during these stages. Human intestinal antimicrobial peptides have been shown to inhibit microsporidian spore germination in cell infection assays^45^. Notably, none of the signal cascades known to be involved in mosquito immune responses appeared to be activated during infection by the two microsporidian species in either *Ae. aegypti* or *An. quadrimaculatus*, including the Toll pathway^46^, broadly induced by diverse pathogens including Gram-positive bacteria, fungi, viruses and *Plasmodium*^47^, the JAK-STAT^48^ pathway that mediates response to dengue virus infection in *Ae. aegypti* or the IMD pathway that regulates antimicrobial immune responses in *Drosophila*^49^. This suggests that even when an immune response is mounted against microsporidian invasion, expression changes, including induction of the MD2-like receptors ML33 and ML15B, do not trigger the same immune cascades as many other pathogens.

Contrary to expectations based on their host ranges, infection of the first generation of *Ae. aegypti* by *E. aedis* elicited a notable immune response from the mosquito, while infection of *An. quadrimaculatus* by *V. culicis* did not. Given the broad host range of *V. culicis*, it could have been expected that the generalist would be less able to evade any specific host’s immune response. One possibility is that *V. culicis* infection involves fewer intercellular movements than infection by *E. aedis*, as infection by *V. culicis* in *An. quadrimaculatus* larvae is not progressive and the developing pathogen does not leave the initially infected tissues until the adult stage^50^. *E. aedis*, on the other hand, travels between a wide variety of host cell types, including oenocytes, potentially exposing it to immune responses. Alternatively, some portion of the differences in the immune responses of *Aedes* and *Anopheles* may reflect the large evolutionary distance between the two genera. The evasion of the mosquito immune response by *E. aedis* when vertically transmitted to *Ae. aegypti* may involve lack of detection of the pathogen when inherited, so that it is not recognized as foreign. In the long-standing association between the intracellular endosymbiont *Wolbachia* and *Drosophila*, *Wolbachia* does not induce host immune gene expression^51^. Alternatively, the microsporidian may be shielded from recognition by the immune system during these phases, since the infections originated in the developing eggs and/or because they are entirely intracellular within fat body. Another possibility is that *E. aedis* may actively suppress the host response through its wide array of secreted proteins.

In summary, the combination of genome comparisons and deep transcriptional profiling of the mosquito hosts and microsporidian pathogens described here illustrate differing evolutionary trajectories for specialist and generalist pathogens and interactions with their hosts. Future studies of additional microsporidian–host systems should reveal whether similar strategies evolve convergently in other Microsporidia. In a broader context, identification of additional proteins involved in mosquito–pathogen interactions contributes to ongoing efforts in developing strategies to prevent transmission of malaria^52^ and potentially other mosquito-vectored pathogens. Furthermore, such studies in invertebrate models may provide parallels for the innate immune response in humans to microsporidian infections^53^.

## Methods

### Strain isolation and genome sequencing

*E. aedis* isolate USNM 41457 was isolated in 1982 from Thailand^54^, and is propagated in *Ae. aegypti* Orlando strain (1952) mosquitoes. *V. culicis* floridensis was isolated during a survey for pathogens between 1991 and 1994 from wild-type *Ae. albopictus* in Florida^36^; the strain is propagated in *An. quadrimaculatus* and *Helicoverpa zea*. To isolate *E. aedis* spores, *Ae. aegypti* larvae vertically infected with *E. aedis* were homogenized in a glass tissue grinder and filtered through a 50-μ mesh. The filtered homogenate was then washed by centrifuging twice at 4,000*g* for 5 min. The resuspended pellet containing the spores was then loaded on a 30% Ludox (Sigma-Aldrich) column with 5 mM NH_4_Cl and centrifuged at 16,000*g* for 10 min. The resuspended pellet was then loaded on a 60% Ludox column with 5 mM NH_4_Cl and centrifuged at 16,000*g* for 10 min, producing a band of *E. aedis* spores at the top of the gradient. Spores were removed and washed by centrifuging twice at 4,000*g* for 15 min in sterile deionized water. *V. culicis* spores were purified from infected adult *H. zea* using continuous Ludox gradients as described in ref. 55.

Genomic DNA was prepared from purified spores by bead-beating followed by DNA isolation using the Omniprep DNA extraction kit (G-Biosciences)^55^. Purity of microsporidian genomic DNA was assessed using qPCR with primer sets specific to both two microsporidian genes and two host genes. All *E. aedis* samples were screened with the *E. aedis* SSU gene (GenBank accession code AF027684.1) sense primer 5′-ACGGGTTCTTATGCTCATGC-3′ and antisense primer 5′-CGGGTGAAGATGTGAGTGTG-3′; the *E. aedis* beta tubulin gene (GenBank accession code 186970548) sense primer 5′-TTTCCAAGGCTGCACTTCTT-3′ and antisense primer 5′-CTCAGCCAGCAAATGTTTGA-3′; the *Ae. aegypti* SSU ribosomal gene (GenBank accession code AY988440.1) sense primer 5′-GCAGCCCAGAACATCTAAGG-3′ and antisense primer 5′-ATACGTCAACCCATCGTGGT-3′; and the *Ae. aegypti* beta-2 tubulin gene (GenBank accession code DQ833526.1) sense primer 5′-TGGTAAGATTTTGGGCAGGT-3′ and antisense primer 5′-ATTTTCTGTCGTTCCTTCGC-3′. All *V. culicis* samples were screened with the *V. culicis* SSU gene (GenBank accession code AJ252961.1) sense primer 5′-GCGAATCCGATGATAAAGGA-3′ and antisense primer 5′-GGGCCCTACTCAATTTCTCC-3′; the *V. culicis* beta tubulin gene sense primer 5′-GCTTGTGATGAGTGGTGTG-3′ and antisense primer 5′-AATGCAATCGTGGAAATGG-3′; the *H. zea* SSU ribosomal gene sense primer 5′-ATGCATGTCTCAGTGCAAGC-3′ and antisense primer 5′-TCATCCCATCACTGGTCAGA-3′; and the *H. zea* beta-2 tubulin gene sense primer 5′-CTTCTGTAGCACAATTCTGCC-3′ and antisense primer 5′-TGCGCTGAACAGCAAATTAC-3′. All qPCR showed >10 cycle difference between microsporidian and host amplicons; therefore, <10% host contamination was determined for each genomic DNA sample before sequencing.

A fragment library and a 3.2-kb insert library were constructed from the genomic DNA for each species and sequenced using 454 GS FLX Titanium sequencing technology (Supplementary Table 11), and the resulting unpaired and paired reads (respectively) were assembled with Newbler (version MapAsmResearch-04/19/2010-patch-08/17/2010). *V. culicis* was assembled with the –het option and screened for insect host using the –versus option for Newbler. Final read depth in the assembly was on average 31X. *E. aedis* was assembled using the -het option and the -versus option to screen out contaminating sequences using a bacterial database of organisms detected in preliminary assemblies. In addition, post assembly, bacterial contamination in the *E. aedis* assembly was removed by filtering contigs with high identity alignments to bacteria in the NCBI nt database, and by removing contigs with markedly different GC content and read depth (GC>50% and depth <8 ×) compared with the assembled *E. aedis* sequence. Final read depth in the assembly was on average 20 × .

### Polymorphism in *E. aedis* and *V. culicis*

The 454 sequence was also analysed for evidence of polymorphism by aligning reads to the assembly using the BWA-SW algorithm^56^ and identifying variants with GATK^57^. As for previous analysis of variation in *Nematocida*, SNPs were identified using the GATK UnifiedGenotyper, and filtered using the GATK VariantFiltration using the version 3 best practices for hard filters (QD<2.0, MQ<40.0, FS>60.0, HaplotypeScore >13.0, MQRankSum<−12.5, ReadPosRankSum<−8.0). SNPs were also filtered requiring a GATK GenotypeQuality ≥30 and read depth ≥10. For both assemblies, alignment statistics were emitted for all sites using UnifiedGenotyper, and used to count callable positions (read depth at least 10 and quality of at least 30) for estimates of SNPs per kb.

### RNA isolation and sequencing

Healthy second instar *Ae. aegypti* larvae were exposed in groups of 100 to *E. aedis* spores from homogenized infected larvae at a dose of 1 × 10^3^ and 1 × 10^4^ spores per ml for 24 h and then transferred in groups of 1,000 into 56 cm × 45 cm × 8 cm plastic rearing trays with 3 l of deionized water. Trays were fed 2 g of 3:2 bovine liver powder:brewer’s yeast slurry every other day until pupation and were reared under standard laboratory conditions (25 °C, 12/12 light cycle, 80% relative humidity). Adults were given 10% sucrose *ad libitum* and bloodfed on bovine blood in sausage casings warmed to 55 °C. At 3 days post exposure, one sample of 75 dissected larval gastric cacea from the group exposed to the higher spore dose was collected (Fig. 1d, time point 1). At 2–3 days post emergence, 25 abdomens from female *Ae. aegypti* exposed to the lower spore dose were dissected (Fig. 1d, time point 2). A bovine blood meal was supplied to the mosquitoes at 2–3 days post emergence, and 24 h post-blood meal 25 abdomens from blood-fed female *Ae. aegypti* were collected (Fig. 1d, time point 3). Next, *Aedes aegypti* eggs collected from infected adults were hatched and reared following standard procedures. Larvae were sampled at 3 days post hatch (Fig. 1d, time point 4) and again at 6 days post hatch (Fig. 1d, time point 5). At each time point, matched tissue samples of healthy *Ae. aegypti* were also taken as controls. The complete infection sequence was replicated twice for all samples. Uninucleate *E. aedis* spores were purified by the same 30 and 60% Ludox gradient method used to obtain purified spores for genomic DNA extraction. Spores were purified from two separate clutches of infected *Ae. aegypti* (1 × 10^8^ and 7 × 10^7^ spores' final yield) for RNA extraction.

To sample *V. culicis*, healthy neonate *An. quadrimaculatus* larvae were exposed to *V. culicis* at a dose of 1 × 10^6^ spores per ml for 24 h and then transferred in groups of 1,000 into 56 cm × 45 cm × 8 cm plastic rearing trays with 3 l of deionized water. Trays were fed 0.5 g of 1:1 bovine liver powder:brewer’s yeast slurry every other day for two feedings and then 0.5 g of 1:1:1 bovine liver powder:brewer’s yeast:potbelly pig chow slurry every other day for two feedings. Rearing occurred under standard laboratory conditions (25 °C, 12/12 light cycle, 80% relative humidity), and adults were given 10% sucrose *ad libitum*. Fourth instar larvae were sampled at 7 days post exposure (Fig. 1e, time point 1). Abdomens from 20 female adults were dissected 3–4 days post emergence (Fig. 1e, time point 2). As with *E. aedis* sampling, uninfected host control samples were taken at each time point. *V. culicis* spores were purified by the same continuous Ludox gradient method used to obtain purified spores for genomic DNA extraction. Spores were purified from infected adult *An. quadrimaculatus* from two separately infected groups (5 × 10^8^ and 1 × 10^10^ spores final yield) for RNA extraction.

Total RNA was isolated from all tissue samples using the RNAqueous-4PCR Kit (Ambion/Life Technologies) or the Quick-RNA MiniPrep kit (Zymo Research) as per the manufacturer’s instructions. Purified *E. aedis* and *V. culicis* spores were pelleted and resuspended in 500 μl of RNA lysis buffer. An equal volume of siliconized glass beads (425–600 μm) was added, and the suspension was mechanically disrupted using the Mini-Beadbeater-1 (BioSpec) at setting 48 (4,800 r.p.m.) for 30 s. Disruption of spores was confirmed with hemacytometer counts. The lysate was removed from the glass beads and processed through RNA extraction. To confirm that microsporidian RNA was successfully extracted, qPCR was performed on cDNA synthesized from total RNA as described for genomic DNA. Strand-specific libraries were constructed for poly(A)-selected RNA samples using the dUTP second strand-marking method^58^,^59^. Libraries were sequenced on an Illumina HiSeq to generate an average of 76 million paired-end reads (101-nt) per sample; all samples had high sequencing quality scores, ranging from an average of 29.2 to 34.6 per sample (Supplementary Table 12).

### Prediction of gene structures and repetitive elements

Gene structures were annotated using a combination of *ab initio* methods and RNA-Seq data. An initial set of protein-coding genes was predicted using Prodigal^60^ and Genewise^61^ run with Uniref90 and available microsporidian protein sets, requiring proteins smaller than 100 amino acids to have good supporting evidence including a PFAM domain^62^ match, nongeneric gene product name assigned or prediction by both methods. The Prodigal model was chosen for each locus and then any nonoverlapping Genewise models were added. Genes overlapping other transcripts including tRNA or rRNA features were manually inspected and edited where possible. Gene corresponding to repetitive elements (matching proteins or PFAM domains found in repetitive elements or with a TPSI hit (requiring expect <1e−10 and >30% coverage)) were removed from the gene set. For *A. algerae*, we included the assembled and annotated genome of PRA139 (AOMW00000000); the previously described genome of a human isolate^16^ was not used, as an assembly but not a gene set was available in NCBI.

Gene structures were updated and validated using RNA-Seq. Reads were assembled using the Inchworm component of Trinity; the resulting transcripts were aligned to the genome with PASA^63^. Cases of candidate merged or novel genes were manually reviewed and corrected where possible, and untranslated repeat sequence supported by these transcripts was added.

Introns were initially identified using the motif described in ref. 64. Furthermore, all *V. culicis* and *E. aedis* RNA-Seq reads were aligned to the genome using TopHat2 (ref. 65) with a minimum and maximum intron size of 10 and 300, respectively. *V. culicis* genes with spliced read mappings that matched the motif predictions were considered to have introns; no *E. aedis* genes had spliced read mappings with aligned mates or predicted motifs. A number of *V. culicis* ribosomal protein genes had motifs but lacked any RNA-Seq read coverage of their 5′ end where the motif occurs; these genes were also considered to have introns. Splicing efficiency was then calculated as the depth of coverage of reads in the intron divided by the depth of coverage of reads adjacent to the intron.

The completeness of all new assemblies was evaluated by examining the conservation and completeness of core eukaryotic genes (CEGs)^15^. We compared our gene set by blast to the CEGMA set, and identified hits above and below the recommended 70% coverage threshold. A tool for streamlined analysis and visualization of conservation of CEGs is available on SourceForge (http://sourceforge.net/projects/corealyze/).

Repetitive elements in each genome were identified using RepeatModeler version open-1.0.7 (http://www.repeatmasker.org/RepeatModeler.html), and these consensus sequences were mapped across each genome using RepeatMasker version open-3.2.8 (http://www.repeatmasker.org).

### Gene clustering and evolutionary analyses

Orthologous gene families were identified for *E. aedis*, *V. culicis*, *A. algerae*, *T. hominis* (ANCC00000000) and 10 Microsporidia, 12 fungi and one choanoflagelate outgroup that had been previously analysed^8^ using OrthoMCL^66^ version 1.4 with an inflation index of 1.5 and maximum *e*-value of 1 × 10^−5^. To estimate a phylogeny, we identified the 217 orthologues present as single copy in all genomes. Amino-acid sequences of these clusters were aligned using MUSCLE^67^ and poorly aligned regions were trimmed with trimAl using the -gappyout option^68^. Individual protein alignments were then concatenated into a single aligned sequence. Next, we utilized ProtTest 3.3 (ref. 69) to determine that LG+gamma was the best-fitting model for the data; the PROTCATLG model was used to infer a phlylogeny using RAxML 7.7.8 (ref. 70) with 1,000 bootstrap replicates.

Genes were placed into metabolic pathways based on their orthologues in *S. cerevisiae*. In addition, to search for more divergent orthologues of spliceosomal proteins, HMMs were constructed from alignments of the fungal and microsporidian orthologues and used to identify additional microsporidian orthologues, using hmmbuild and hmmsearch in HMMER3 (hmmer.org) and requiring that new orthologues have a match of at least 1e−10, are the best hit, and are not mapped orthologues of other *S. cerevisiae* genes. Similarly, presence of RNAi machinery was determined by the presence of orthologues of Argonaute and Dicer (as in ref. 27). Secreted proteins of unknown function (candidate effector proteins) were identified by finding proteins that were (a) predicted to be secreted by SignalP version 4 (ref. 71), (b) lacked any known domains and (c) were expressed (≥1 FPKM) in at least one condition.

### Transcription analysis

RNA-Seq reads were aligned to Microsporidian transcript sequences using Bowtie^72^. Transcript abundance was estimated using RSEM^73^, TMM-normalized FPKM for each transcript were calculated and differentially expressed transcripts were identified using edgeR^74^, all as implemented in the Trinity package version r2013-2-25 (ref. 75). To determine correlations between samples, FPKM values were log-transformed and then Pearson’s correlation coefficients were calculated; the biological replicates of all samples were highly correlated, ranging from 0.89 to 0.98 in *V. culicis* and from 0.92 to 0.98 in *E. aedis*,(Fig. 3a, Supplementary Fig. 9). Plotting the coverage of RNA-Seq reads across transcripts of both microsporidia (from 5′ to 3′) showed that reads are evenly represented across transcript length (Supplementary Fig. 10). To identify GO (http://geneontology.org/) term enrichment of differentially expressed genes, we used YeastMine^76^ with yeast orthologues as input. Additional enrichment tests for pathways, candidate effectors, surface proteins, gene families and other features were conducted using Fisher’s exact test.

*Ae. aegypti* transcripts (version 1.4) were downloaded from VectorBase (www.vectorbase.org)^77^. A set of 18,724 *An. quadrimaculatus* Orlando (1952) transcripts was assembled using the Trinity package from the combined RNA-Seq generated from nine different life stages of the Orlando strain (eggs, 1st instar, 2nd instar, 3rd instar, 4th instar, early pupae, late pupae, adult female and adult male), and was used as a reference gene set for this species. Alignment of RNA-Seq reads and calculation of transcript abundance were conducted as described above. To identify the GO term and KEGG pathway enrichment of differentially expressed genes, we classified transcripts using Blast2GO^78^ and KAAS^79^, and then performed comparisons with Fisher’s exact test. We also identified mosquito immune genes using ImmunoDB^34^; for *Ae. aegypti* we used the available annotations, while for *An. quadrimaculatus* we used OrthoMCL^66^ to map to the *An. gambiae* transcriptome version 4.1 (downloaded from VectorBase) and then transferred the annotations from *An. gambiae.*

## Additional information

**Accession codes:** The sequence data from this study have been deposited in the NCBI BioProject database under accession code PRJNA210750. Assemblies were submitted under accession codes AEUG01000000 for *V. culicis*, AFBI01000000 for *E. aedis* and GBTE01000000 for the *An. quadrimaculatus* transcriptome.

**How to cite this article:** Desjardins, C.A. *et al.* Contrasting host-pathogen interactions and genome evolution in two generalist and specialist microsporidian pathogens of mosquitoes. *Nat. Commun.* 6:7121 doi: 10.1038/ncomms8121 (2015).

## Supplementary Material

Supplementary Information Supplementary Figures 1-10, Supplementary Tables 1-12, Supplementary Notes 1-7 and Supplementary References

Supplementary Data 1 Conservation of spliceosomal proteins.

Supplementary Data 2 Gene clusters matching phylogenetic profile of microsporidia with splicing.

Supplementary Data 3 Expression values of *E. aedis* RNA-Seq samples.

Supplementary Data 4 Expression values of *V. culicis* RNA-Seq samples.

Supplementary Data 5 Conservation of core meiotic genes across microsporidia genomes.

Supplementary Data 6 Expression values of *Ae. aegypti* RNA-Seq samples.

Supplementary Data 7 Expression values of *An. quadrimaculatis* RNA-Seq samples.

## Figures and Tables

**Table 1:** Genome statistics of *E. aedis* and *V. culicis*.

	***E. aedis***	***V. culicis***
Genome size (Mb)	51.3	6.1
Scaffolds	1,429	379
Scaffold N50 (kb)	434	94
GC content (%)	22.5	39.7
GC content coding (%)	29.8	42.7
Repeats (%)	5.5	4.2
Low complexity (%)	12.1	0.4
Coding regions (%)	9	47
Genes	4,190	2,773
Paralogues	722	226
Spliced genes	0	27
RNA-Seq-supported genes (FPKM≥1)	3,631	2,535
Predicted surface proteins	517	282
Candidate effectors*	335	169

^*^Defined as secreted proteins with no known domains that are expressed during infection.

**Table 2:** GO term enrichment in infected and control mosquitoes*.

**GO term**	**Up in**	***q*** **-value**
*Ae. aegypti* time point 1 (larval midgut)		
Alkaline phosphatase activity [GO:0004035]	Control	8.22E−07
Structural constituent of cuticle [GO:0042302]	Control	2.09E−06
Magnesium ion binding [GO:0000287]	Control	5.33E−03
Metabolic process [GO:0008152]	Control	3.56E−02
Iron ion transport [GO:0006826]	Infected	2.14E−02
Cellular iron ion homeostasis [GO:0006879]	Infected	2.14E−02
Ferric iron binding [GO:0008199]	Infected	2.14E−02
Polysaccharide binding [GO:0030247]	Infected	2.69E−02
Endopeptidase inhibitor activity [GO:0004866]	Infected	3.78E−02
		
*Ae. aegypti* time point 2 (early adult oenocyte)		
Proteolysis [GO:0006508]	Control	4.39E−08
Serine-type endopeptidase activity [GO:0004252]	Control	7.92E−08
Digestion [GO:0007586]	Control	1.87E−07
Binding [GO:0005488]	Control	4.46E−03
Phototransduction [GO:0007602]	Control	1.82E−02
Serine-type endopeptidase activity [GO:0004252]	Infected	2.10E−11
Proteolysis [GO:0006508]	Infected	4.66E−10
Lysozyme activity [GO:0003796]	Infected	3.18E−03
Carbohydrate binding [GO:0030246]	Infected	9.58E−03
rRNA N-glycosylase activity [GO:0030598]	Infected	9.74E−03
		
*Ae. aegypti* time point 3 (late adult oenocyte)		
Extracellular space [GO:0005615]	Control	2.87E−04
Serine-type endopeptidase inhibitor activity [GO:0004867]	Control	2.87E−04
Copper ion binding [GO:0005507]	Control	1.76E−03
Laccase activity [GO:0008471]	Control	7.64E−04
Oocyte development [GO:0048599]	Control	8.85E−04
Serine-type endopeptidase activity [GO:0004252]	Infected	5.66E−30
Proteolysis [GO:0006508]	Infected	3.01E−23
Endopeptidase inhibitor activity [GO:0004866]	Infected	7.98E−03
Sugar binding [GO:0005529]	Infected	4.52E−02
		
*Ae. aegypti* time point 4 (early larval fat body)		
None		
		
*Ae. aegypti* time point 5 (late larval fat body)		
Dynein complex [GO:0030286]	Control	7.02E−06
Microtubule-based movement [GO:0007018]	Control	8.99E−04
Nucleosome assembly [GO:0006334]	Control	1.50E−03
Microtubule motor activity [GO:0003777]	Control	3.13E−03
Axoneme [GO:0005930]	Control	3.35E−02
Structural constituent of cuticle [GO:0042302]	Infected	4.25E−11
		
*An. quadrimaculatus* time point 1 (larval systemic)		
none		
		
*An. quadrimaculatus* time point 2 (adult systemic)		
Ryanodine-sensitive calcium-release channel activity [GO:0005219]	Infected	8.56E−4
Muscle contraction [GO:0006936]	Infected	2.01E−3
Calcium ion binding [GO:0005509]	Infected	5.68E−3
Cellular calcium ion homeostasis [GO:0006874]	Infected	1.11E−2
Striated muscle thick filament [GO:0005863]	Infected	1.14E−2

GO, gene ontology.

^*^Mosquito developmental stage and site of infection are listed for each time point. The top five significantly enriched terms in control and infected samples at each time point are shown for each species (*q*<0.05, Fisher’s exact test). No significantly enriched GO terms were found for *Ae. aegypti* time point 4 or *An. quadrimaculatus* time point 1.
