# Supplementary material for: Contrasting host–pathogen interactions and genome evolution in two generalist and specialist microsporidian pathogens of mosquitoes
Source: Nat Commun. 2015 May 13;6:7121. doi: 10.1038/ncomms8121 (PMC4435813; doi:10.1038/ncomms8121)
Supplement: Supplementary Information — Supplementary Figures 1-10, Supplementary Tables 1-12, Supplementary Notes 1-7 and Supplementary References [file ncomms8121-s1.pdf]

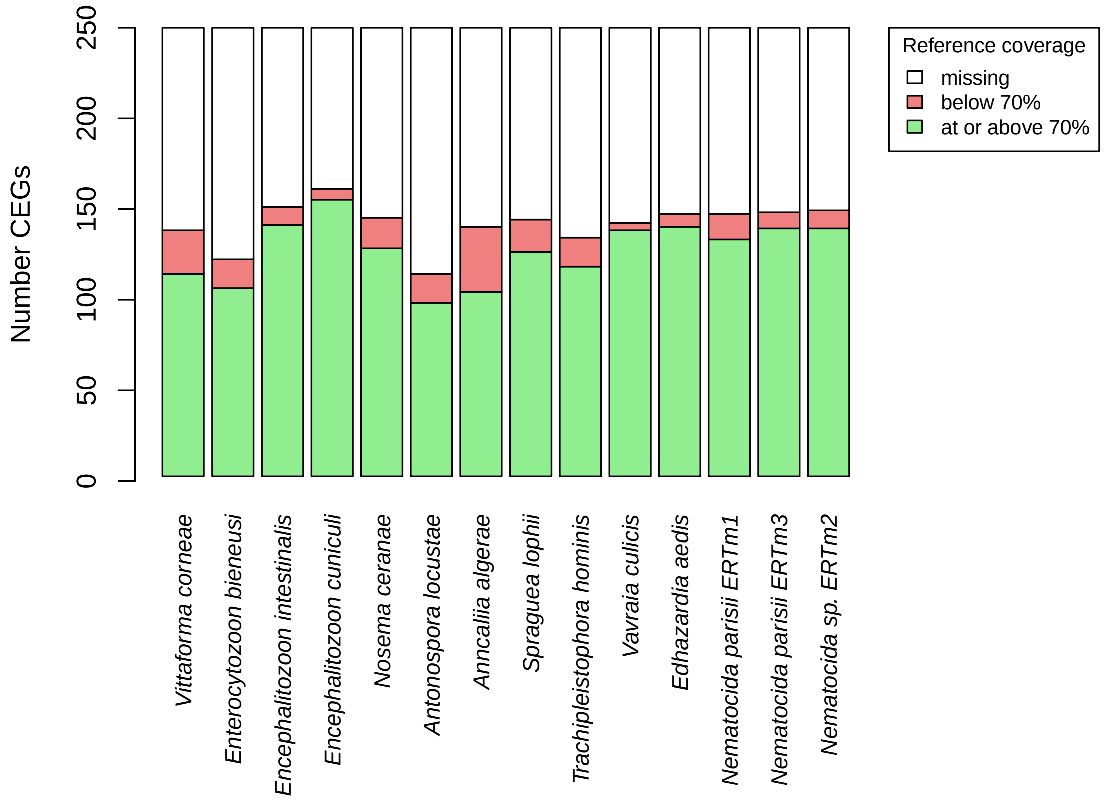

Supplementary Figure 1. Conservation of core eukaryotic gene (CEG) set across Microsporidia genomes. The percent coverage of genes with significant Blast similarity is shown for alignments above and below the recommended 70% coverage threshold, which can indicate partial gene structures.

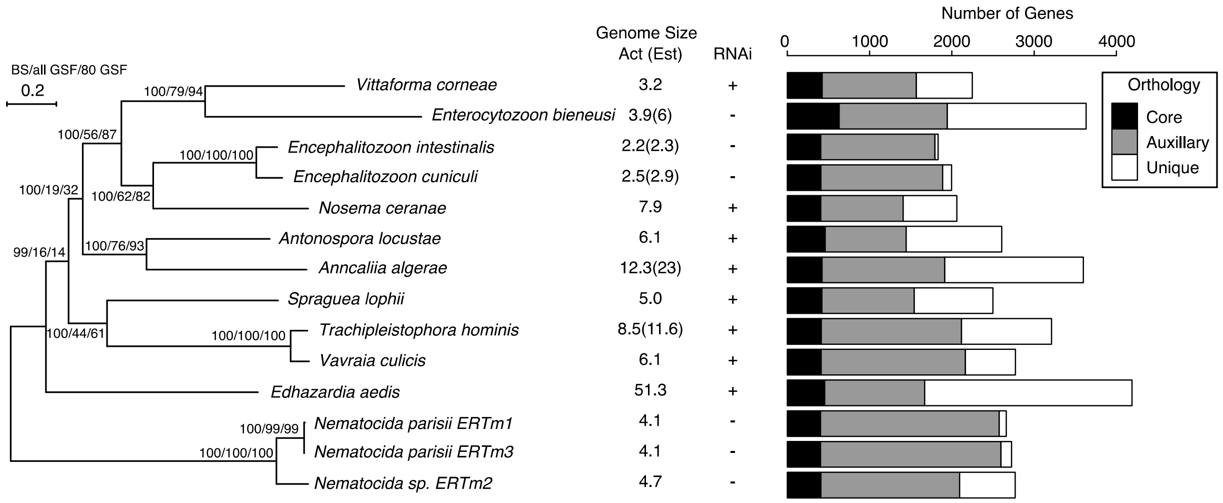

Supplementary Figure 2. Phylogeny of Microsporidia based on 217 single copy core genes. The phylogeny was estimated using RAxML [1] and a PROTCATLG model of evolution. Bootstrap support (BS) and gene support frequency (all GSF) is shown above each node. In an attempt to increase GSF, we re-estimated the phylogeny with the 71 genes with an average bootstrap value of 80 or higher. The resulting phylogeny was identical in topology and bootstrap support to the original tree, and showed increased GSF (80 GSF) across most nodes. Genome size (as actual and estimated), presence or absence of RNAi machinery, and ortholog distribution is also shown (core, in all genomes; auxillary, in two or more genomes; unique, in one genome).

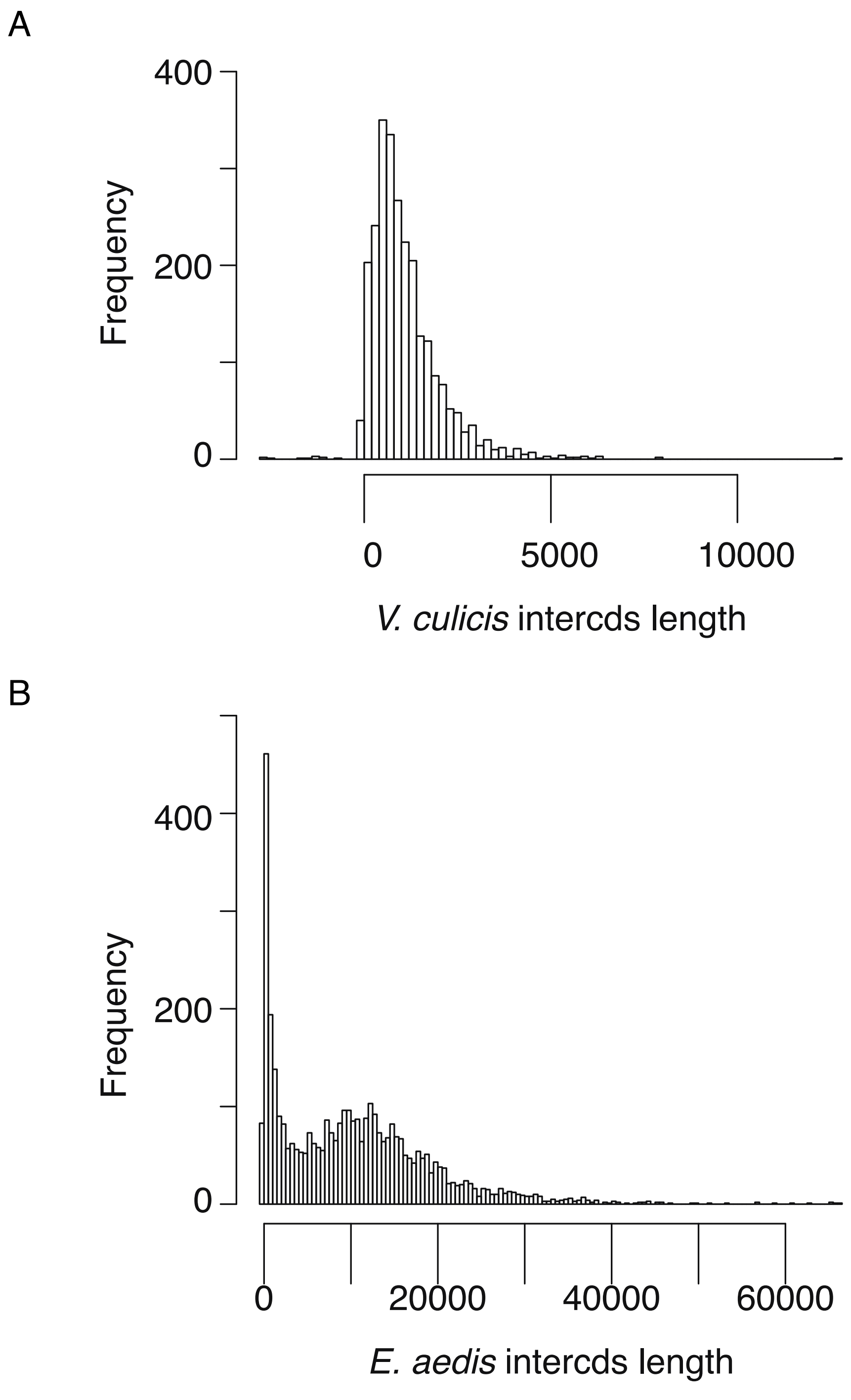

Supplementary Figure 3. Intergenic region distribution for *E. aedis* and *V. culicis*. The distribution of regions length between the start and stop of adjacent CDS is shown for A. *V. culicis* and B. *E. aedis*.

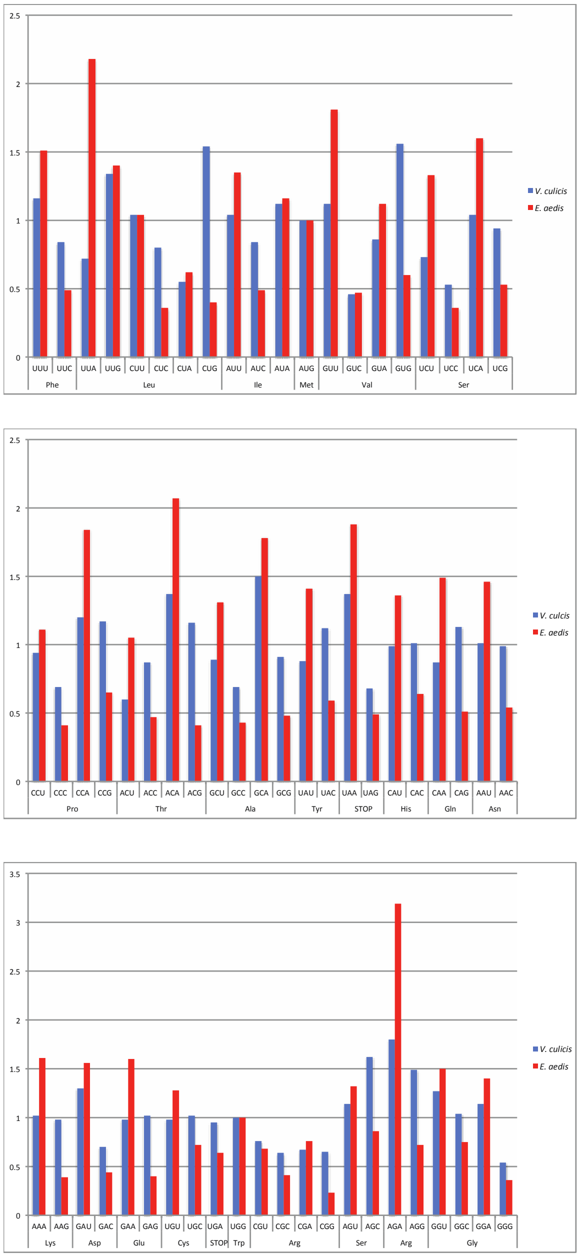

Supplementary Figure 4. Codon usage in *E. aedis* and *V. culicis* protein coding genes. The normalized relative usage for codons for the same amino acid is plotted for each species.

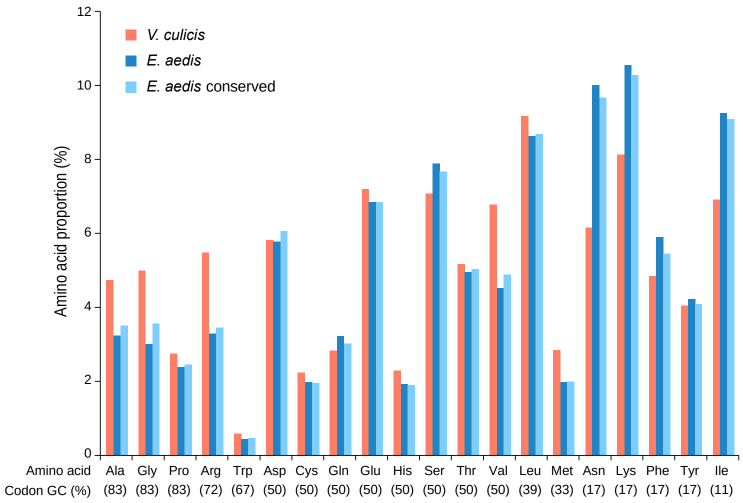

Supplementary Figure 5. Bias in amino acid usage in *E. aedis*. The overall proportion of different amino acids found in protein coding genes was computed for *E. aedis* (blue) and *V. culicis* (orange); for *E. aedis* both the set of all genes (dark blue) or the subset of genes conserved in at least one other species (light blue) were used for comparison. Amino acids are sorted left to right based on the average GC content of the corresponding codons, listed in the bottom row.

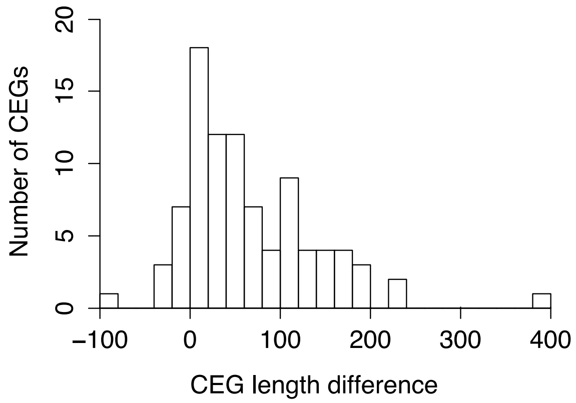

Supplementary Figure 6. Histogram of length differences between 91 orthologous CEGs (core eukaryotic genes) in Microsporidia and Fungi. Differences were calculates as the average amino acid length of the fungal CEG minus the average amino acid length of the microsporidial CEG. Fungi have significantly longer CEGs than Microsporidia ($p < 1.68e-13$; Wilcoxon signed-rank test).

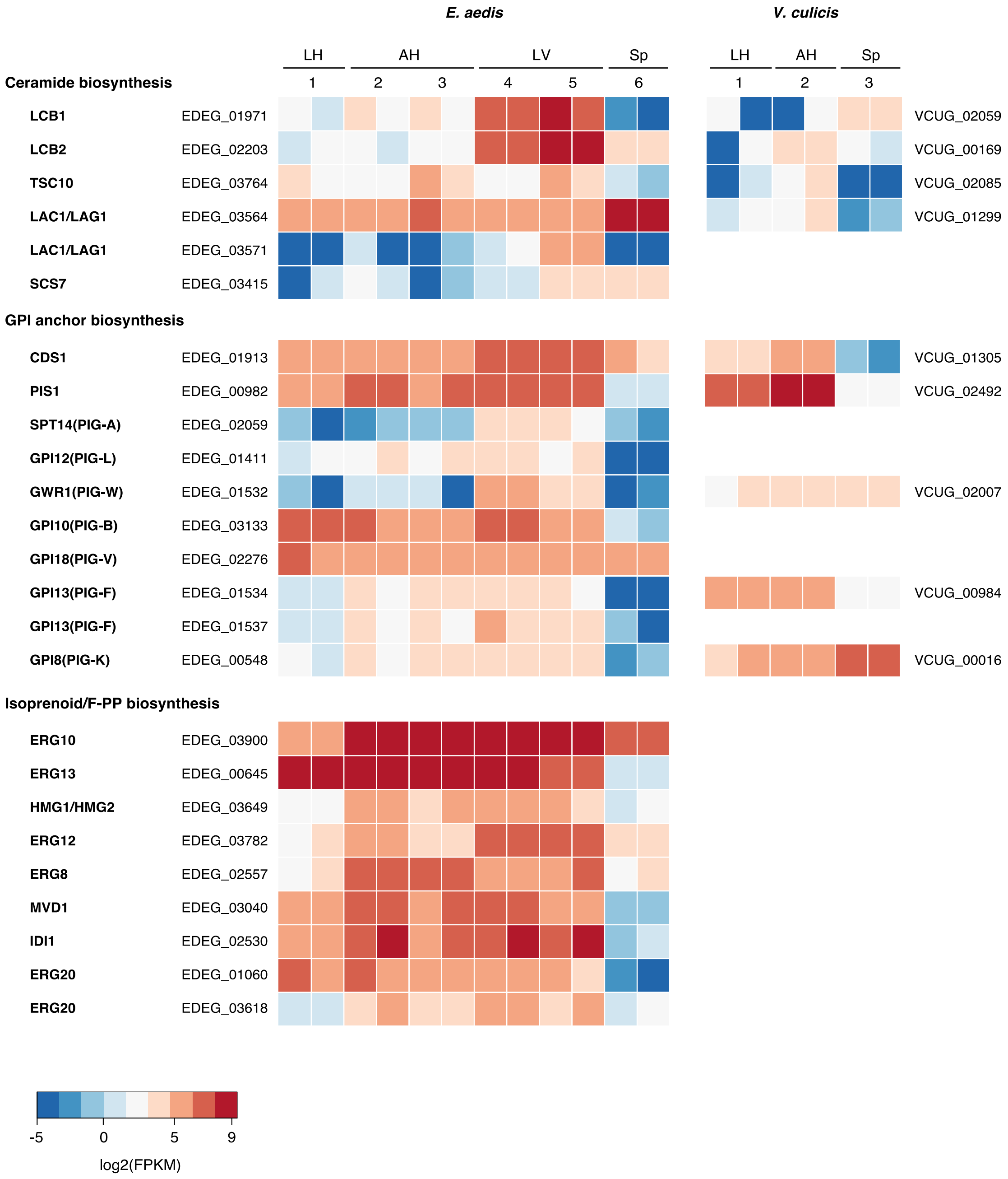

Supplementary Figure 7. Differential expression of metabolic pathways involved in signaling and cell membrane/protein decoration. Pathways shown include ceramide (sphingolipid) biosynthesis, GPI anchor biosynthesis, and isoprenoid biosynthesis. Absence of a row in *V. culicis* indicates that no ortholog exists for that gene. Sampling points are numbered as shown in Figure 1. LH: larval horizontal; AH: adult horizontal; LV: larval vertical; Sp: isolated spores.

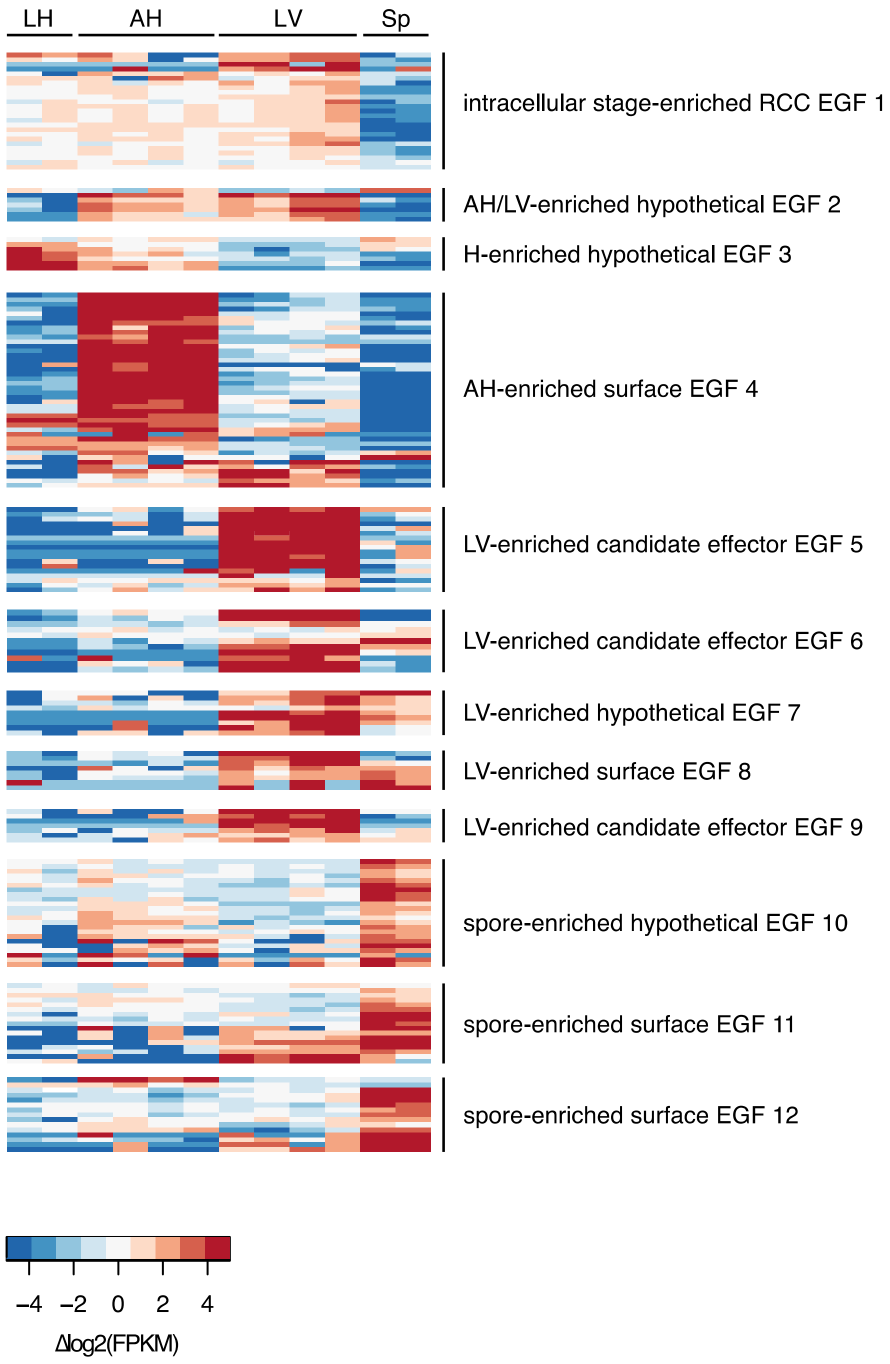

Supplementary Figure 8. Differentially expressed expanded gene families (EGFs) in *E. aedis*. Gene families are named after their phase of enrichment followed by their functional annotations. LH: larval horizontal; AH: adult horizontal; LV: larval vertical; Sp: isolated spores. Colors correspond to the change from the median in log-transformed FPKM.

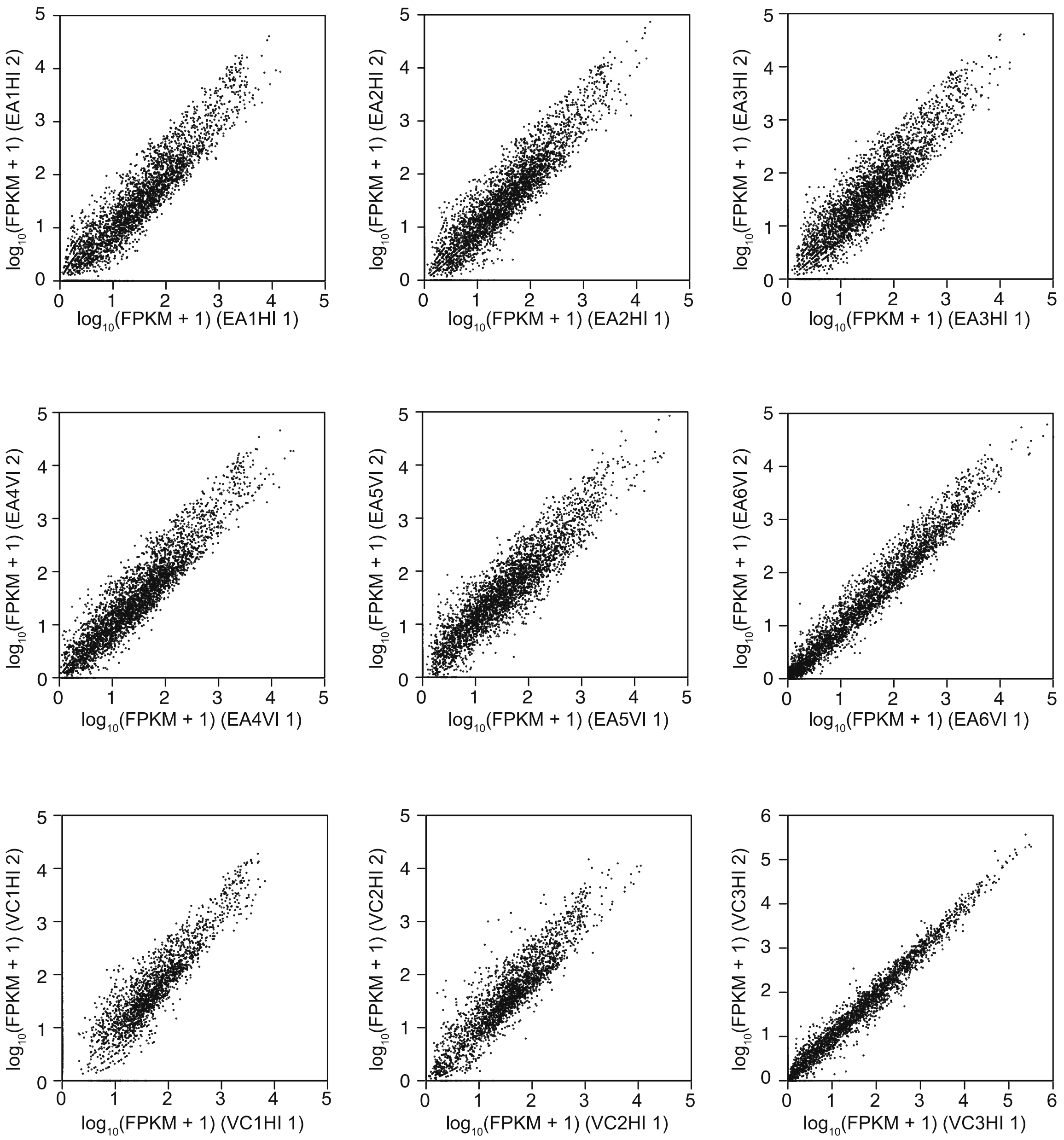

Supplementary Figure 9. Comparison of transcript FPKM values for biological replicates of *E. aedis* and *V. culicis*. To give more equal weight to genes with lower expression values, the $\log_{10}$ values of the FPKM + 1 for each gene are plotted. Numbering of *E. aedis* (EA) and *V. culicis* (VC) samples is as described in the main text (Fig. 1); for each horizontal (H) or vertical (V) stage the two biological replicates are compared.

A

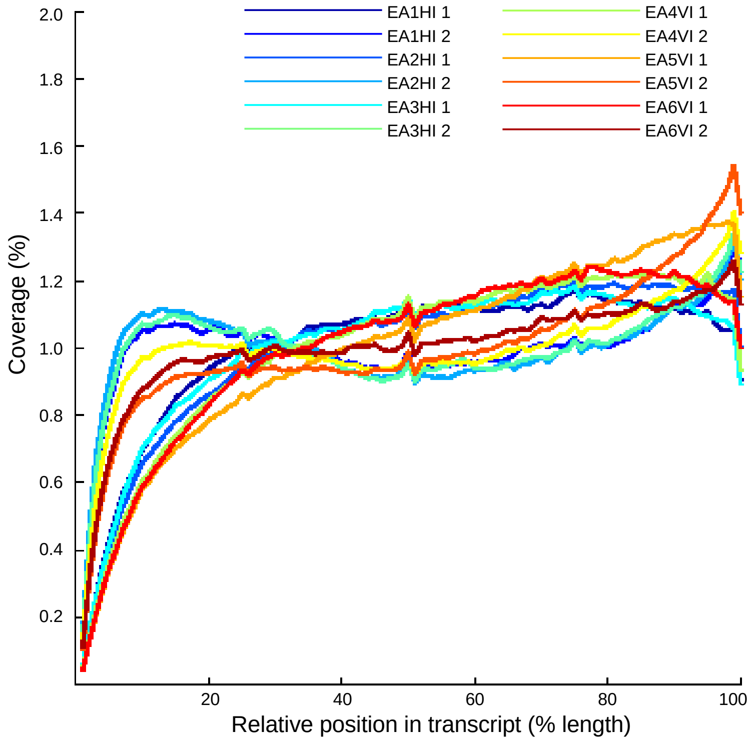

B

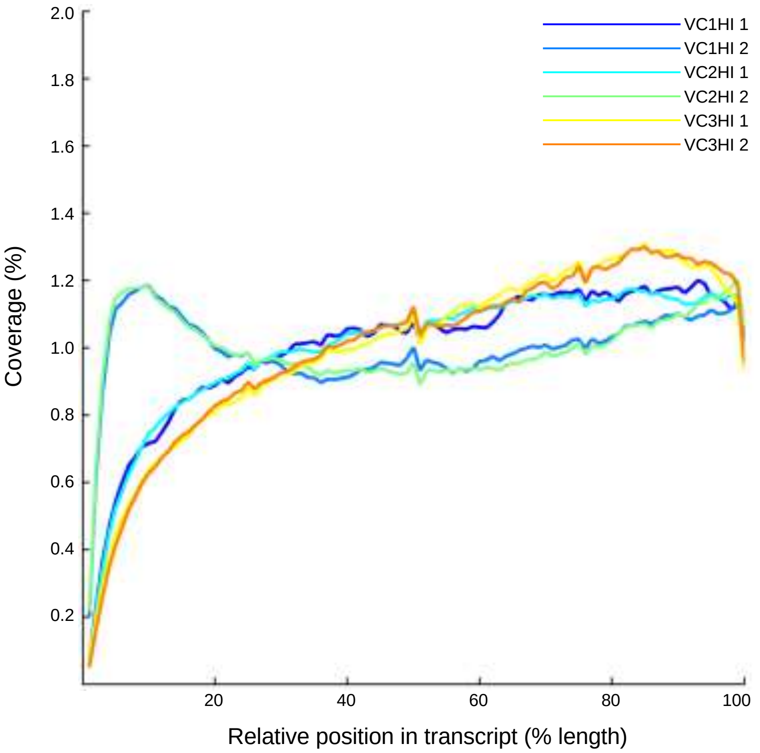

Supplementary Figure 10. Transcript coverage for RNA-Seq samples. Normalized coverage of RNA-Seq samples aligned to microsporidia transcripts is plotted at the relative position of transcript length for *E. aedis* and *V. culicis* samples (A, B).

Supplementary Table 1. *V. culicis* predicted introns. For each intron, the gene id, the name or function, the intron location (5’ end, 3’ end, or mid-gene), intron size, and percent of RNA-Seq data that was spliced for that intron (N/A values represent introns without RNA-Seq coverage) is provided. Multiple rows for a single gene indicate multiple introns in that gene. Genes combined with a plus indicate genes that were modeled as two intronless genes, but based on intron identification represent a single spliced gene.

Gene ID	Gene Name / Function	Intron Location	Intron Size (bases)	Percent Spliced
VCUG_00040	surface	3'	23	N/A
VCUG_00092	PFAM:Sedlin_N	3'	23	26.9
VCUG_00161	surface	3'	24	N/A
VCUG_00234	PFAM:ThiF	5'	22	51.4
VCUG_00244 + VCUG_02834	surface	mid	22	50
VCUG_00244 + VCUG_02834	surface	mid	22	11.1
VCUG_00376	cold shock	mid	24	79.2
VCUG_00403	S23		69	69
VCUG_00509	surface	3'	19	N/A
VCUG_00771	L18	5'	23	74.3
VCUG_00792	peptidyl-tRNA hydrolase	mid	22	27.7
VCUG_00981	surface	mid	22	15.6
VCUG_00982	surface	mid	23	21.5
VCUG_00982	surface	mid	22	25.6
VCUG_00983	surface	mid	23	18.4
VCUG_00983	surface	mid	22	17.6
VCUG_01015	S26	5'	25	N/A
VCUG_01407	surface	3'	22	19.4
VCUG_01487	S27	5'	28	N/A
VCUG_02062	L24	5'	28	N/A
VCUG_02065	S17	5'	25	N/A
VCUG_02256 + VCUG_02257	surface	mid	23	14.3
VCUG_02405	surface	mid	23	5.7
VCUG_02406	surface	mid	23	25
VCUG_02406	surface	mid	22	18.2
VCUG_02407	surface	mid	23	23.7
VCUG_02407	surface	mid	22	26.7
VCUG_02408	surface	mid	22	9.9
VCUG_02408	surface	mid	23	29.6
VCUG_02506	L44	5’*	38	N/A
VCUG_02547	L1	5'	26	87.7
unmodeled	L39	5'	25	N/A
unmodeled	surface	mid	22	35.1

*This intron has the motif, but with 15bp in the region near the 3’ splice site previously described as being constrained to 3bp [2].

Supplementary Table 2. Top 20 enriched GO terms in genes upregulated in *V. culicis* environmental spores. Starred values were significant at a 0.05 FDR.

GO Term	P-value
RNA processing [GO:0006396]	5.05E-07*
organic cyclic compound metabolic process [GO:1901360]	5.99E-07*
nucleobase-containing compound metabolic process [GO:0006139]	6.78E-07*
RNA metabolic process [GO:0016070]	9.15E-07*
cellular aromatic compound metabolic process [GO:0006725]	9.90E-07*
heterocycle metabolic process [GO:0046483]	9.90E-07*
nucleic acid metabolic process [GO:0090304]	2.21E-06*
gene expression [GO:0010467]	5.11E-06*
ncRNA processing [GO:0034470]	6.66E-06*
cellular nitrogen compound metabolic process [GO:0034641]	8.21E-06*
rRNA processing [GO:0006364]	1.18E-05*
nitrogen compound metabolic process [GO:0006807]	2.25E-05*
rRNA metabolic process [GO:0016072]	9.09E-05*
ribonucleoprotein complex biogenesis [GO:0022613]	1.20E-04*
ncRNA metabolic process [GO:0034660]	2.59E-04*
cellular component organization or biogenesis [GO:0071840]	8.55E-04
chromatin organization [GO:0006325]	0.001181
ATP-dependent chromatin remodeling [GO:0043044]	0.001292
nucleosome organization [GO:0034728]	0.001375
chromatin modification [GO:0016568]	0.001479

Supplementary Table 3. Top 20 enriched GO terms in genes upregulated in *E. aedis* environmental spores. Starred values were significant at a 0.05 FDR.

GO Term	P-value
ncRNA processing [GO:0034470]	1.20E-07*
rRNA processing [GO:0006364]	3.70E-07*
rRNA metabolic process [GO:0016072]	3.50E-06*
ribosome biogenesis [GO:0042254]	4.32E-06*
ribonucleoprotein complex biogenesis [GO:0022613]	1.47E-05*
RNA processing [GO:0006396]	7.87E-05*
maturation of SSU-rRNA [GO:0030490]	1.87E-04
ncRNA metabolic process [GO:0034660]	2.31E-04
maturation of SSU-rRNA from tricistronic rRNA transcript [GO:0000462]	2.47E-04
ribosomal small subunit biogenesis [GO:0042274]	3.08E-04
maturation of LSU-rRNA [GO:0000470]	5.73E-04
maturation of LSU-rRNA from tricistronic rRNA transcript [GO:0000463]	0.001796
ribosomal large subunit biogenesis [GO:0042273]	0.002274
maturation of 5.8S rRNA [GO:0000460]	0.003626
maturation of 5.8S rRNA from tricistronic rRNA transcript [GO:0000466]	0.003626
nucleobase-containing compound metabolic process [GO:0006139]	0.004454
RNA modification [GO:0009451]	0.005254
cellular aromatic compound metabolic process [GO:0006725]	0.006512
heterocycle metabolic process [GO:0046483]	0.006512
cellular component biogenesis [GO:0044085]	0.007526

Supplementary Table 4. Top 20 enriched GO terms in genes upregulated in *V. culicis* intracellular stages. Starred values were significant at a 0.05 FDR.

GO Term	P-value
single-organism biosynthetic process [GO:0044711]	5.35E-06*
carbohydrate metabolic process [GO:0005975]	7.04E-05
monosaccharide metabolic process [GO:0005996]	7.61E-05
single-organism carbohydrate metabolic process [GO:0044723]	1.32E-04
hexose metabolic process [GO:0019318]	1.88E-04
protein processing [GO:0016485]	2.85E-04
glucose metabolic process [GO:0006006]	4.56E-04
protein maturation [GO:0051604]	6.90E-04
carbohydrate catabolic process [GO:0016052]	7.84E-04
single-organism carbohydrate catabolic process [GO:0044724]	7.84E-04
cofactor metabolic process [GO:0051186]	9.03E-04
cellular protein complex assembly [GO:0043623]	0.00205
DNA strand elongation [GO:0022616]	0.002115
lagging strand elongation [GO:0006273]	0.002586
organophosphate biosynthetic process [GO:0090407]	0.002627
coenzyme metabolic process [GO:0006732]	0.003941
glycerophospholipid biosynthetic process [GO:0046474]	0.003941
organonitrogen compound metabolic process [GO:1901564]	0.004211
pyridine nucleotide metabolic process [GO:0019362]	0.004383
nicotinamide nucleotide metabolic process [GO:0046496]	0.004383

Supplementary Table 5. Top 20 enriched GO terms in genes upregulated in *E. aedis* intracellular stages.

GO Term	P-value
proteasomal protein catabolic process [GO:0010499]	1.06E-04
vacuolar transport [GO:0007034]	3.45E-04
lipid metabolic process [GO:0006629]	0.001041
cellular protein complex assembly [GO:0043623]	0.001052
catabolic process [GO:0009056]	0.001678
cellular lipid metabolic process [GO:0044255]	0.001749
isoprenoid metabolic process [GO:0006720]	0.00176
isoprenoid biosynthetic process [GO:0008299]	0.00176
proteolysis [GO:0006508]	0.001976
lipid biosynthetic process [GO:0008610]	0.00234
protein lipidation [GO:0006497]	0.002387
phosphatidylinositol biosynthetic process [GO:0006661]	0.002387
lipoprotein metabolic process [GO:0042157]	0.002387
lipoprotein biosynthetic process [GO:0042158]	0.002387
endomembrane system organization [GO:0010256]	0.002675
GPI anchor metabolic process [GO:0006505]	0.002814
GPI anchor biosynthetic process [GO:0006506]	0.002814
glycolipid metabolic process [GO:0006664]	0.002814
steroid metabolic process [GO:0008202]	0.002814
glycolipid biosynthetic process [GO:0009247]	0.002814

Supplementary Table 6. Top 20 enriched GO terms in genes upregulated in *E. aedis* vertically inherited intracellular stages vs. horizontally inherited stages in host larvae. Starred values were significant at a 0.05 FDR.

GO Term	P-value
cellular lipid metabolic process [GO:0044255]	5.65E-06*
lipid metabolic process [GO:0006629]	1.45E-05*
lipid biosynthetic process [GO:0008610]	1.94E-05*
membrane lipid biosynthetic process [GO:0046467]	2.69E-05*
membrane lipid metabolic process [GO:0006643]	4.33E-05*
single-organism biosynthetic process [GO:0044711]	6.54E-05*
protein lipidation [GO:0006497]	7.60E-05*
lipoprotein metabolic process [GO:0042157]	7.60E-05*
lipoprotein biosynthetic process [GO:0042158]	7.60E-05*
generation of precursor metabolites and energy [GO:0006091]	1.01E-04*
carbon catabolite activation of transcription from RNA polymerase II promoter [GO:0000436]	2.76E-04
regulation of cellular respiration [GO:0043457]	2.76E-04
regulation of generation of precursor metabolites and energy [GO:0043467]	2.76E-04
carbon catabolite activation of transcription [GO:0045991]	2.76E-04
organophosphate biosynthetic process [GO:0090407]	2.94E-04
single-organism process [GO:0044699]	3.29E-04
phospholipid metabolic process [GO:0006644]	6.56E-04
phospholipid biosynthetic process [GO:0008654]	7.08E-04
carbon catabolite regulation of transcription from RNA polymerase II promoter [GO:0000429]	0.001053
carbon catabolite regulation of transcription [GO:0045990]	0.001053

Supplementary Table 7. Top 20 enriched GO terms in genes upregulated in *E. aedis* vertically inherited intracellular stages vs. horizontally inherited stages in host adults. Starred values were significant at a 0.05 FDR.

GO Term	P-value
single-organism process [GO:0044699]	4.18E-07*
single-organism cellular process [GO:0044763]	4.57E-06*
response to chemical stimulus [GO:0042221]	2.93E-05*
single-organism biosynthetic process [GO:0044711]	3.97E-05*
cell communication [GO:0007154]	7.70E-05*
generation of precursor metabolites and energy [GO:0006091]	8.22E-05*
response to organic substance [GO:0010033]	1.73E-04
organophosphate biosynthetic process [GO:0090407]	1.78E-04
phosphorus metabolic process [GO:0006793]	1.80E-04
membrane lipid biosynthetic process [GO:0046467]	1.81E-04
membrane lipid metabolic process [GO:0006643]	2.87E-04
sulfur compound metabolic process [GO:0006790]	6.37E-04
regulation of signal transduction [GO:0009966]	6.44E-04
regulation of signaling [GO:0023051]	6.44E-04
carbon catabolite activation of transcription from RNA polymerase II promoter [GO:0000436]	6.55E-04
regulation of cellular respiration [GO:0043457]	6.55E-04
regulation of generation of precursor metabolites and energy [GO:0043467]	6.55E-04
carbon catabolite activation of transcription [GO:0045991]	6.55E-04
nucleotide-sugar biosynthetic process [GO:0009226]	7.22E-04
signaling [GO:0023052]	7.24E-04

Supplementary Table 8. Counts of differentially expressed genes in comparisons of infected versus control mosquitoes. Counts are based on FDR < 0.05.

Comparison	Up in infected	Up in control
A. aegypti-E. aedis		
1 (LH)	63	52
2 (AH)	118	28
3 (AH)	193	16
4 (LV)	2	4
5 (LV)	39	119
A. quadrimaculatus-V. culicis		
1 (LH)	5	0
2 (AH)	90	0

Supplementary Table 9. KEGG pathway enrichment in infected and control mosquitoes. Mosquito developmental stage and site of infection are listed for each timepoint. All significantly enriched terms in control and infected samples at each time point are shown for each species (q < 0.05, Fisher's exact test).

KEGG Pathway	Representative Genes	Up in	q-value
***Ae. aegypti* timepoint 1 (larval midgut)**			
ko00627|Aminobenzoate degradation	alkaline phosphatases	control	5.88E-04
ko00790|Folate biosynthesis	alkaline phosphatases	control	5.88E-04
ko02020|Two-component system	alkaline phosphatases	control	5.88E-04
***Ae. aegypti* timepoint 2 (early adult oenocyte)**			
none			
***Ae. aegypti* timepoint 3 (late adult oenocyte)**			
none			
***Ae. aegypti* timepoint 4 (early larval fat body)**			
none			
***Ae. aegypti* timepoint 5 (late larval fat body)**			
ko05016|Huntington's disease	dynein	control	1.31E-03
***An. quadrimaculatus* timepoint 1 (larval systemic)**			
none			
***An. quadrimaculatus* timepoint 2 (adult systemic)**			
ko05412|Arrhythmogenic right ventricular cardiomyopathy	actinin, ryanodine receptors	control	6.52E-04
ko04260|Cardiac muscle contraction	tropomyosin, ryanodine receptors	control	1.34E-03
ko05410|Hypertrophic cardiomyopathy	tropomyosin, ryanodine receptors	control	1.34E-03
ko05414|Dilated cardiomyopathy	tropomyosin, ryanodine receptors	control	1.34E-03
ko04261|Adrenergic signaling in cardiomyocytes	tropomyosin, ryanodine receptors	control	1.11E-02

Supplementary Table 10. Glycolytic genes in *Ae. aegypti*.

Gene ID	Gene name
AAEL009387	hexokinase
AAEL012994	glucose-6-phosphate isomerase
AAEL004295	glucose-6-phosphatase
AAEL010590	aldose-1-epimerase
AAEL007315,AAEL0152091	glucose-6-phosphate 1-epimerase
AAEL001158	fructose-1,6-bisphosphatase
AAEL006895	phosphofructokinase
AAEL005766	fructose-bisphosphate aldolase
AAEL001480,AAEL010037	phosphoglucomutase
AAEL004988	phosphoglycerate kinase
AAEL002542	triosephosphate isomerase
AAEL006070	phosphoglycerate mutase
AAEL001668	enolase
AAEL011421	multiple inositol polyphosphate phosphatase
AAEL009507	glucose-6-phosphate 1-dehydrogenase
AAEL004434	transketolase
AAEL009389	transaldolase
AAEL003246	deoxyribose-phosphate aldolase

Supplementary Table 11. Sequencing reads generated using 454 Technology.

	Fragment Library		3 kb Library	
	Reads	Bases	Reads	Bases
E. aedis	2,533,129	837,636,402	1,195,684	391,556,598
V. culicis	460,882	142,253,805	238,340	79,854,666

Supplementary Table 12. RNA-Seq read statistics and accession codes.

Sample	SRA Accession code	Total Reads	PF Reads*	PF Reads (%)	Average quality
E. aedis EA1HC	SRX390041	68,481,002	63,489,682	92.7%	31.69
E. aedis EA1HC	SRX737086	87,486,894	81,161,346	92.8%	32.87
E. aedis EA1HI	SRX390044	45,766,066	42,369,178	92.6%	31.54
E. aedis EA1HI	SRX737092	81,974,890	75,920,986	92.6%	32.93
E. aedis EA2HC	SRX390045	50,533,250	46,908,810	92.8%	31.91
E. aedis EA2HC	SRX737091	81,825,558	75,965,252	92.8%	32.73
E. aedis EA2HI	SRX390046	56,393,016	52,281,814	92.7%	31.87
E. aedis EA2HI	SRX737082	90,429,928	83,621,422	92.5%	32.62
E. aedis EA3HC	SRX390047	58,319,732	53,705,024	92.1%	31.13
E. aedis EA3HC	SRX737083	76,927,998	70,691,960	91.9%	32.28
E. aedis EA3HI	SRX390048	73,127,460	67,563,176	92.4%	31.42
E. aedis EA3HI	SRX737085	80,626,540	74,571,242	92.5%	32.65
E. aedis EA4VC	SRX734310	67,297,884	62,375,296	92.7%	31.66
E. aedis EA4VC	SRX736586; SRX736585	80,417,528	74,058,262	92.1%	32.32
E. aedis EA4VI	SRX390049	62,088,858	57,444,536	92.5%	31.72
E. aedis EA4VI	SRX737084	78,049,988	72,567,846	93.0%	33.14
E. aedis EA5VC	SRX390050	73,361,274	67,938,544	92.6%	31.38
E. aedis EA5VC	SRX737093	82,231,070	76,588,864	93.1%	32.75
E. aedis EA5VI	SRX390051	83,944,952	77,871,896	92.8%	32.02
E. aedis EA5VI	SRX737088	85,566,354	79,363,336	92.8%	32.80
E. aedis EA6V	SRX390052	81,002,448	75,548,002	93.3%	31.74
E. aedis EA6V	SRX737094	83,917,140	78,633,420	93.7%	34.04
V. culicis VC1HC	SRX390056	63,192,238	57,984,960	91.8%	30.52
V. culicis VC1HC	SRX709711; SRX709708	65,141,412	58,906,006	90.4%	29.23
V. culicis VC1HI	SRX390057	68,314,474	62,700,460	91.8%	30.01
V. culicis VC1HI	SRX709709; SRX709704	86,111,938	78,678,462	91.4%	31.16
V. culicis VC2HC	SRX390058	61,729,818	56,497,816	91.5%	30.32
V. culicis VC2HC	SRX709712; SRX709705	75,560,562	69,398,664	91.8%	31.53
V. culicis VC2HI	SRX390059	57,613,564	52,927,206	91.9%	30.96
V. culicis VC2HI	SRX709710; SRX709707	75,204,770	69,149,806	91.9%	31.62
V. culicis VC3H	SRX390055; SRX390054; SRX390053	72,151,970	69,529,002	96.4%	34.62
V. culicis VC3H	SRX470848; SRX465842	88,756,302	79,192,556	89.2%	33.93

***Illumina Passing Filter (PF).**

Supplementary Note 1: AT-rich expansion and codon/amino acid usage bias in *E. aedis*

Both genes and repetitive sequences are distributed across the *E. aedis* assembly, with no large regions absent of genes or repeats. The lengths of intergenic regions in *E. aedis* follow a bimodal distribution, in which most pairs of genes are separated by large intergenic regions but some have remained closely linked (**Supplementary Fig. 3**).

Comparing the relative codon usage for a given amino acid for *E. aedis* and *V. culicis*, AT-rich codon frequencies are increased and the GC-rich codon frequencies are decreased (**Supplementary Fig. 4, Supplementary Fig. 5**). This AT-bias has also impacted the frequencies of amino acids found in proteins. In *E. aedis* proteins, amino acids with the highest average GC of their codons are under-represented while amino acids with the lowest average GC of their codons are over-represented, even when only conserved genes are considered (**Supplementary Fig. 5**). A similar bias in amino acid usage has been previously reported for other AT-rich genomes [3].

Supplementary Note 2: Phylogenetic position of *E. aedis*

While all nodes in this phylogeny displayed high bootstrap support, analysis of individual gene trees uncovered discrepant branching order around some tree nodes. We therefore calculated gene support frequencies (GSF) [4], i.e. the percentage of individual core gene trees that supported each node in the tree, using RAxML to estimate trees for individual single copy core genes under the same model as the concatenated gene tree. This revealed a low GSF at the base of the branch to *E. aedis.* Removing genes with low phylogenetic signal from the analysis (gene trees with bootstrap support < 80) and re-estimating the phylogeny with the 71 well supported genes, as suggested in ref.[4] did not improve support for the placement of *E. aedis* (**Supplementary Fig. 2**). This calls into question the phylogenetic placement of this species and suggests that more taxa may be needed to fully resolve the basal branching order. The genome-wide low GC content likely contributes to the difficulty in robustly placing *E. aedis* on the microsporidian phylogeny.

Supplementary Note 3: Genome compaction

Another strategy for genome compaction is the reduction of protein length, as previously shown the genes in *Encephalitizoon cuniculi*, *Enterocytozoon bienusi*, and *Octospora bayeri* compared to fungal orthologs [5,6]. Here, analysis of core eukaryotic genes (CEGs, [7]) revealed more broadly

that Microsporidia CEGs overall have shorter coding sequences than their fungal orthologs. We compared 91 CEGs found in most of our microsporidian and fungal genomes and found that fungal CEGs are significantly longer than microsporidian CEGs ($p < 1.68e-13$; Wilcoxon signed-rank test). The average difference is 66.1 +/- 72.6 amino acids, and a histogram of these differences is shown in **Supplementary Fig. 6**. This suggests that not only are there evolutionary constraints on microsporidian gene content and intron structure, but coding sequencing length as well.

Supplementary Note 4: Pathway loss in *E. aedis* and *V. culicis*

Genome compact has also occurred by loss of metabolic pathways, though different species have lost or retained different pathways. The *V. culicis* genome has lost five genes the GPI anchor biosynthesis pathway, leaving only *GPI8*, *GPI13*, and *GWT1*; in addition, the GPI anchor synthesis gene *GPI10* has been lost in all Microsporidia except *E. aedis* and *Nematocida*. It is unclear whether these reduced complements of GPI anchor synthesis genes allow *V. culicis* to manufacture GPI anchors, or if these genes have acquired some functional redundancy in another pathway. The *V. culicis* genome also encodes a reduced sphingolipid biosynthesis pathway relative to *E. aedis* (**Fig. 2A**). The *V. culicis* genome encodes a single ceramide synthase as the final step of this pathway, while the *E. aedis* genome encodes two ceramide synthases (paralogs of each other), and also the next enzyme in the pathway, *SCS7* desaturase (along with its *CYB5* cofactor), which allows for the production of more complex ceramides. The *V. culicis* genome does encodes phosphatidylserine decarboxylase, which is involved in the synthesis of aminophospholipids, whereas the *E. aedis* genome lacks this pathway. The phylogenetic distribution of the isoprenoid pathway is nearly the opposite of phosphatidylserine decarboxylase (**Fig. 2A**), and these two pathways may in some way encode some functional redundancy. Outside of metabolism, the *E. aedis* genome encodes three genes (*SSU72*, *ESS1*, and *SPO14*)) involved in Ser5 and Ser7 phosphorylation of RNA polymerase II that are all missing from the *V. culicis* genome.

Supplementary Note 5: Spliced genes in *V. culicis*

While introns are conserved in similar genes across different Microsporidia species, RNA-Seq reveals that introns are not excised in many transcripts in *V. culicis*. The 27 spliced genes identified in *V. culicis* include eight ribosomal protein genes (RPGs), 15 genes with transmembrane domains, and four other genes with known domains (**Supplementary Table 1**). RPG introns, typically found near the 5’ end of genes, included orthologs of the RPGs identified

as spliced in the *T. hominis* genome [8] plus the L1 RPG. Spliced genes with transmembrane domains had introns in the middle portion of the gene, and six of the genes contained two introns each separated by an 11 base exon. Splicing of these introns was variable; there were RNA-Seq based transcripts with no introns spliced out, both introns spliced out, and either the first or second transcript spliced. Intron retention produces a heavily truncated protein, suggesting alternative splicing does not produce variation in cell surface proteins. As in *En. cuniculi* [9], the majority of *V. culicis* genes in all three functional classes were inefficiently spliced (median efficiency = 25.3%, **Supplementary Table 1**); the differential splicing of surface proteins may simply result from this inefficiency.

Supplementary Note 6: Phylogenetic profiling of proteins associated with splicing
To search for genes encoding other potential pre-mRNA splicing factors in Microsporidia, we searched for genes whose phylogenetic profile matched species with verified splicing. Genes matching this profile are found in at least 5 of the 9 Microsporidia with splicing but are absent in all the species without splicing (**Supplementary Data 2**). Of the 29 gene clusters that matched this pattern, 13 were broadly conserved with other fungi while 16 were largely specific to Microsporidia. Eleven genes also conserved among fungi are known components of the spliceosome, including Sm proteins (D1, D2, D3, and F), U2 snRNP proteins (Cus1, Hsh155, Prp9, and Prp11), a U1 snRNP protein (Luc7) and other splicing factors (Bud31, Yju2). Of the 16 other clusters enriched in or specific to Microsporidia, two are involved in polyadenylation. In addition, given that the majority of genes shared with Fungi are known to be involved in splicing, some fraction of the Microsporidia-specific set, including 10 clusters without assigned function, could represent proteins involved in Microsporidia-specific splicing functions.

Supplementary Note 7: Gene-level examination of pathway enrichment in intracellular stages of *E. aedis*
We conducted gene level examination of pathways identified as being enriched in intracellular stages of *E. aedis*. This revealed that GPI anchor construction and isoprenoid synthesis components were nearly universally upregulated in intracellular stages (**Supplementary Fig. 7**). *CYB5*, a cofactor in isoprenoid biosynthesis, was also significantly upregulated ($q < 1.2e-5$). Furthermore, of the five COPI subunits identified in *E. aedis* (*COP1*, *RET3*, *SEC21*, *SEC26*, *SEC27*), all were significantly upregulated in intracellular stages. COPI normally plays a role in Golgi-to-ER transport, and potentially intra-Golgi transport, although a study of *A. locustae* showed COPI and COPII vesicles were not formed [10]. However, among Microsporidia *A.*

locustae uniquely lacks much of the COPII machinery based on our ortholog analysis; this leaves open the possibility that COPI and COPII function differently in *A. locustae* than other Microsporidia. In addition to potential roles in trafficking proteins for secretion, upregulation of COPI machinery may be related to spore production, as the Golgi and ER play a central role in microsporidian spore morphogenesis and/or cell structure, as microsporidian spores lack Golgi [11].

Supplementary References

1. Stamatakis, A. RAxML-VI-HPC: maximum likelihood-based phylogenetic analyses with thousands of taxa and mixed models. *Bioinformatics* **22,** 2688–90 (2006).
2. Lee, R. C. H., Gill, E. E., Roy, S. W. & Fast, N. M. Constrained intron structures in a microsporidian. *Mol. Biol. Evol.* **27,** 1979–1982 (2010).
3. Singer, G. A. C. & Hickey, D. A. Nucleotide Bias Causes a Genomewide Bias in the Amino Acid Composition of Proteins. *Mol. Biol. Evol.* **17,** 1581–1588 (2000).
4. Salichos, L. & Rokas, A. Inferring ancient divergences requires genes with strong phylogenetic signals. *Nature* **497,** 327–331 (2013).
5. Katinka, M. D. *et al.* Genome sequence and gene compaction of the eukaryote parasite *Encephalitozoon cuniculi*. *Nature* **414,** 450–3 (2001).
6. Corradi, N., Haag, K. L., Pombert, J.-F., Ebert, D. & Keeling, P. J. Draft genome sequence of the *Daphnia* pathogen *Octosporea bayer*i: insights into the gene content of a large microsporidian genome and a model for host-parasite interactions. *Genome Biol.* **10,** R106 (2009).
7. Parra, G., Bradnam, K. & Korf, I. CEGMA: a pipeline to accurately annotate core genes in eukaryotic genomes. *Bioinforma. Oxf. Engl.* **23,** 1061–1067 (2007).
8. Heinz, E. *et al.* The genome of the obligate intracellular parasite *Trachipleistophora hominis*: new insights into microsporidian genome dynamics and reductive evolution. *PLoS Pathog.* **8,** e1002979 (2012).
9. Grisdale, C. J., Bowers, L. C., Didier, E. S. & Fast, N. M. Transcriptome analysis of the parasite *Encephalitozoon cunicul*i: an in-depth examination of pre-mRNA splicing in a reduced eukaryote. *BMC Genomics* **14,** 207 (2013).
10. Beznoussenko, G. V. *et al.* Analogs of the Golgi complex in microsporidia: structure and avesicular mechanisms of function. *J. Cell Sci.* **120,** 1288–1298 (2007).
11. Vávra, J. & Larsson, J. I. in *Microsporidia: Pathogens of Opportunity* (eds. Weiss, L. M. & Becnel, J. J.) 1–70 (John Wiley and Sons, 2014).
